# Extracellular Vesicular Analysis of Glypican 1 mRNA and Protein for Pancreatic Cancer Diagnosis and Prognosis

**DOI:** 10.1002/advs.202306373

**Published:** 2024-01-10

**Authors:** Hong Li, Chi‐Ling Chiang, Kwang Joo Kwak, Xinyu Wang, Sital Doddi, Lakshmi V. Ramanathan, Sun M. Cho, Ya‐Chin Hou, Tai‐Shan Cheng, Xiaokui Mo, Yueh‐Shih Chang, Hui‐Lan Chang, Weiming Cheng, Wei‐Ni Tsai, Luong T. H. Nguyen, Junjie Pan, Yifan Ma, Xilal Y. Rima, Jingjing Zhang, Eduardo Reategui, Yeh‐Shiu Chu, Peter Mu‐Hsin Chang, Pei‐Hung Chang, Chi‐Ying F. Huang, Cheng‐Hsu Wang, Yan‐Shen Shan, Chung‐Pin Li, Martin Fleisher, L. James Lee

**Affiliations:** ^1^ Department of Chemical and Biomolecular Engineering Ohio State University Columbus OH 43210 USA; ^2^ Spot Biosystems Ltd. Columbus OH 43212 USA; ^3^ Department of Laboratory Medicine Memorial Sloan Kettering Cancer Center New York NY 10065 USA; ^4^ Institute of Clinical Medicine College of Medicine National Cheng Kung University Tainan 70101 Taiwan; ^5^ Division of General Surgery Department of Surgery National Cheng Kung University Hospital College of Medicine National Cheng Kung University Tainan 70101 Taiwan; ^6^ Institute of Biopharmaceutical Sciences National Yang Ming Chiao Tung University Taipei 11221 Taiwan; ^7^ Center for Biostatistics Ohio State University Columbus OH 43210 USA; ^8^ School of Traditional Chinese Medicine College of Medicine Chang Gung University Taoyuan 33302 Taiwan; ^9^ Division of Hematology‐Oncology Department of Internal Medicine Chang Gung Memorial Hospital Keelung 20401 Taiwan; ^10^ Institute of Clinical Medicine National Yang Ming Chiao Tung University Taipei 11221 Taiwan; ^11^ Division of Gastroenterology and Hepatology Department of Medicine Taipei Veterans General Hospital Taipei 11217 Taiwan; ^12^ Therapeutic and Research Center of Pancreatic Cancer Taipei Veterans General Hospital Taipei 11217 Taiwan; ^13^ Division of Clinical Skills Training Department of Medical Education Taipei Veterans General Hospital Taipei 11217 Taiwan; ^14^ School of Medicine College of Medicine National Yang Ming Chiao Tung University Taipei 11221 Taiwan; ^15^ Department of Biomedical Engineering Ohio State University Columbus OH 43210 USA; ^16^ Brain Research Center National Yang Ming Chiao Tung University Taipei 11221 Taiwan; ^17^ Department of Oncology Taipei Veterans General Hospital Taipei 11217 Taiwan

**Keywords:** Glypican 1 mRNA in exosomes and protein in tumor‐associated microvesicles as a dual biomarker, immune lipoplex nanoparticle biochip assay, PDAC screening and chemotherapy prognosis, single extracellular vesicle analysis

## Abstract

Detecting pancreatic duct adenocarcinoma (PDAC) in its early stages and predicting late‐stage patient prognosis undergoing chemotherapy is challenging. This work shows that the activation of specific oncogenes leads to elevated expression of mRNAs and their corresponding proteins in extracellular vesicles (EVs) circulating in blood. Utilizing an immune lipoplex nanoparticle (ILN) biochip assay, these findings demonstrate that glypican 1 (GPC1) mRNA expression in the exosomes‐rich (Exo) EV subpopulation and GPC1 membrane protein (mProtein) expression in the microvesicles‐rich (MV) EV subpopulation, particularly the tumor associated microvesicles (tMV), served as a viable biomarker for PDAC. A combined analysis effectively discriminated early‐stage PDAC patients from benign pancreatic diseases and healthy donors in sizable clinical from multiple hospitals. Furthermore, among late‐stage PDAC patients undergoing chemotherapy, lower GPC1 tMV‐mProtein and Exo‐mRNA expression before treatment correlated significantly with prolonged overall survival. These findings underscore the potential of vesicular GPC1 expression for early PDAC screenings and chemotherapy prognosis.

## Graphical abstract

Detecting pancreatic duct adenocarcinoma (PDAC) is challenging. Utilizing an immune lipoplex nanoparticle (ILN) biochip assay, these findings show that combined glypican 1 (GPC1) mRNA expression in exosomes and GPC1 membrane protein expression in tumor associated microvesicles can effectively discriminated PDAC cases from benign pancreatic diseases and healthy donors. Lower vesicular GPC1 expressions before chemotherapy correlate significantly with prolonged overall survival.

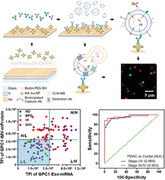

## 1 Introduction

Pancreatic cancer exhibits an exceptionally low 5‐year survival rate of <10% with pancreatic ductal adenocarcinoma (PDAC) as the most prevalent type.^[^ ^1^ ^]^ The high mortality rate associated with pancreatic cancer can be attributed partly to the absence of early detection methods.^[^ ^2^, ^3^ ^]^ Early‐stage pancreatic cancer patients experience limited symptoms, and conventional imaging techniques like X‐ray and CAT scans often lack the required sensitivity and conclusiveness. On the other hand, tissue biopsy is invasive and can sometimes be challenging due to tumor location. Currently, the primary molecular diagnosis method in clinical practice for PDAC involves assessing blood‐based protein biomarkers like carbohydrate antigen 19‐9 (CA19‐9).^[^ ^4^ ^]^ Nevertheless, PDAC patients who lack the Lewis blood group antigen genotype are incapable of generating CA19‐9 antigen in the presence of malignant tissue.^[^ ^5^ ^]^ Additionally, the production of CA19‐9 can vary in levels in patients who possess the Lewis antigen genotype. The variability in expression is the reason why serial measurements of CA19‐9 can only assist in managing a subset of PDAC patients.

In PDAC tumor tissues, mutated driver genes such as *KRAS* and *TP53*, along with their translated proteins, can be identified and measured in cancer cells. While highly specific, obtaining tumor tissues from PDAC patients poses challenges due to the invasiveness of the procedure. Circulating tumor cells (CTCs) and circulating tumor DNA (ctDNA) are being investigated as liquid biopsy biomarkers for cancer diagnosis,^[^ ^6^, ^7^ ^]^ but their low concentration in blood and high cost of detection raises concerns regarding their clinical utility in early cancer screening and treatment prognosis. Extracellular RNAs and proteins have demonstrated stability in blood and other body fluids, partly due to their encapsulation within cell‐secreted extracellular vesicles (EVs) like exosomes and microvesicles.^[^ ^8^, ^9^, ^10^, ^11^ ^]^ Capturing EVs and quantifying the enclosed RNAs and proteins of interest represent a promising strategy for developing non‐invasive assays in cancer detection.^[^ ^12^, ^13^, ^14^, ^15^ ^]^

Glypicans are heparan sulfate proteoglycans anchored to the outer plasma membrane via a glycosyl‐phosphatidylinositol linkage. Their primary role involves regulating the Wnts, Hedgehogs, fibroblast growth factors, and bone morphogenetic protein signaling pathways.^[^ ^16^ ^]^ Glypican‐1 (GPC1) expression is notably elevated in various cancer types, including PDAC,^[^ ^15^ ^]^ breast cancer,^[^ ^17^ ^]^ esophageal squamous cell carcinoma,^[^ ^18^ ^]^ glioblastoma,^[^ ^19^ ^]^ colorectal cancer,^[^ ^20^ ^]^ hepatocellular carcinoma,^[^ ^21^ ^]^ and cervical cancer.^[^ ^22^ ^]^ Transfecting GPC1 RNAi has demonstrated the ability to impede the mitogenic response in cultured colorectal cancer cells,^[^ ^23^ ^]^ establishing GPC1 as a potential biomarker for cancer detection.

Exosomal GPC1 protein exhibited promise for both early‐ and late‐stage detection of PDAC patients.^[^ ^24^ ^]^ However, replicating these high specificities and sensitivities has been non‐trivial.^[^ ^15^, ^25^, ^26^, ^27^ ^]^ We established the effectiveness of exosomal GPC1 mRNA as a superior biomarker for early PDAC detection using a hairpin DNA circuit within cationic lipid‐polymer hybrid nanoparticles (LPHNs).^[^ ^28^ ^]^ However, the challenge with this technique lies in precisely encapsulating the four components required to achieve signal amplification within nanosized LPHNs. Furthermore, the exosomal GPC1 protein study by Melo et al.^[^ ^24^ ^]^ and the GPC1 mRNA detection in our previous study^[^ ^28^ ^]^ employed the time‐consuming ultracentrifugation method for EV isolation, which is not practical for clinical applications.

Therefore, we introduce an Immune Lipoplex Nanoparticle (ILN) biochip assay designed to capture specific EV subpopulations and detect the contents of GPC1 mRNA and their corresponding translated protein at the individual EV level. To isolate EVs from liquid samples, we employed fast, facile, and commercially available EV isolation kits. Our investigation revealed that the exosome‐dominated EV subpopulation (Exo), captured using a combination of CD63/CD9/CD81 antibodies, proved effective as an mRNA‐based cancer marker. Conversely, tumor‐associated microvesicles (tMV), captured through a blend of antibodies targeting antigens abundant on the cancer cell surface, were identified as suitable as a membrane protein (mProtein)‐based cancer marker.

Through the ILN biochip assay, we successfully validated the GPC1 Exo‐mRNA/GPC1 tMV‐mProtein biomarker concept for PDAC. To assess the translational potential of the ILN biochip assay and the expression of GPC1 Exo‐mRNA/tMV‐mProtein in early PDAC screening and treatment prognosis, we conducted comprehensive evaluations using substantial clinical patient samples collected from multiple hospitals in both the US and Taiwan. Additionally, we compared our EV‐GPC1 biomarker performance against levels of CA19‐9, the established clinical standard biomarker for PDAC diagnosis and prognosis. The combined GPC1 Exo‐mRNA/GPC1 tMV‐mProtein expression, together with CA19‐9 levels in blood, effectively distinguished early‐ and late‐stage PDAC patients from healthy donors and benign pancreatic diseases (BPD). Furthermore, our EV analysis demonstrated a strong correlation between the expression of GPC1 Exo‐mRNA/GPC1 tMV‐mProtein before chemotherapy and the overall survival of late‐stage PDAC patients. This correlation was not achievable through the measurement of CA19‐9 levels in blood, indicating the prognostic potential of our assay.

## 2 Results

### 2.1 ILN Biochip for Single EV Capture and Detection with High Sensitivity

**Figure 1:**
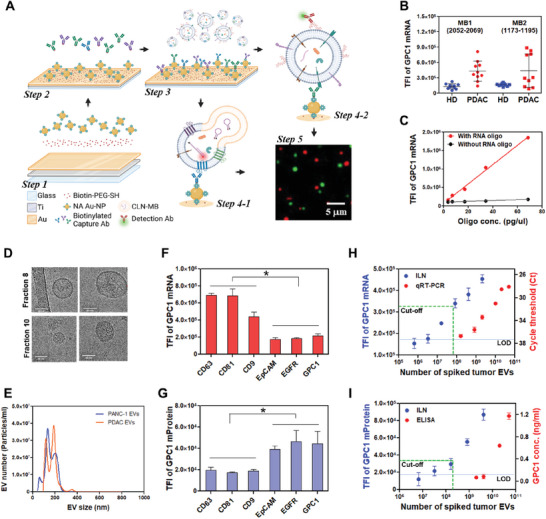
Design of the ILN biochip assay for vesicular GPC1 expression. A) Schematic of the ILN biochip assay. Total Internal Reflection Fluorescence (TIRF) microscopy images were enlarged from 80 µm × 80 µm to 20 µm × 20 µm to show bright spots. B) GPC1 mRNA expression in EVs was verified by two MB designs recognizing different local sequences in GPC1 mRNA. One (2052‐2069, NM_002081.3) showed slightly better discrimination between 10 healthy individuals (Zen‐bio Inc.) and 10 PDAC patient samples (OSU) than the other (1173‐1195, NM_002081.3). C) The performance of MB (2052‐2069, (NM_002081.3) was confirmed by the serial dilution of synthetic standard vesicles with or without a synthetic RNA oligo as a GPC1 mRNA mimic. D) Transmission electron microscopy (TEM) images showed large and small EVs in a PDAC patient serum sorted by size exclusion chromatography (SEC, qEV column, 70 nm), Scale bar, 50 nm. E) NanoSight nanoparticle tracking analysis on EVs from conditioned media of PANC‐1 cells (Blue) or serum of a PDAC patient (Orange) showed two sized groups with mean diameters of 125 and 200 nm, respectively. The EV concentration was ranged in 1E7 particles per mL. F) PANC‐1cell‐derived EVs were captured by different antibodies and vesicular GPC1 mRNA expression was measured by the ILN biochip. Vesicular GPC1 mRNA showed higher expression in the exosome‐dominated EV subpopulation (captured by CD63, CD81, or CD9 antibody). G) Vesicular GPC1 protein showed higher expression in the EV subpopulations captured by PDAC‐associated antibodies (EpCAM/EGFR/GPC1). Calibration curves of EV GPC1 H) mRNA and I) mProtein expression in PANC‐1 cell‐derived EVs spiked into healthy donor serum in comparison with qRT‐PCR and ELISA, respectively. Both limit of detection (LOD) and cut‐off values to distinguish PDAC from control are marked. TFI; Total Fluorescence Intensity. Data were presented as means ± SD (*n* = 2 wells, each well with 100 images). *p* values were determined by the two‐way ANOVA test. *p < 0.05.

The concept of a thin titanium/gold (Ti/Au) coated ILN biochip assay depicted in **Figure** **1**A was designed to perform liquid biopsies based on single‐EV analysis, requiring only a small quantity of clinical samples. A glass coverslip, coated with a nanometer layer of Ti/Au, was initially treated with a linker solution, of 20‐tetradecyloxy‐3,6,7,12,15,18, 22‐heptaoxahexa‐tricontane‐1‐thiol (WC14)^[^ ^29^ ^]^ and biotin‐PEG‐SH in β‐mercaptoethanol. The coverslip was then affixed to a plastic spacer featuring an array of 4 mm wells (Figure S1, Supporting Information). To construct the ILN biochip assay, 40 nm neutravidin gold nanoparticles were introduced and anchored onto the chip surface through biotin‐neutravidin interactions (**Step 1**). Subsequently, biotin‐conjugated capture antibodies were employed to adhere to the surface of these gold nanoparticles, facilitating the capture of a selected EV subpopulation from a liquid sample. The utilization of conjugated gold nanoparticles served to diminish background signals and enhance the discernible differences between healthy donors and patient samples^[^ ^30^ ^]^ (Figure S2A, Supporting Information). **Step 2** illustrates the immobilization of antibodies or cocktail of antibodies (Abs) on a surface‐functionalized glass coverslip, which constitutes the ILN biochip. **Step 3** demonstrates the capture of specific EV subpopulations from a purified body fluid using the tethered antibodies. After washing, **Step 4‐1** exhibits the detection of a target RNA within the captured EV subpopulation using molecular beacon (MB) probes encapsulated within cationic lipoplex nanoparticles (CLNs). These CLN‐MB particles featured a mean particle size of 151.4 ± 7.8 nm, a zeta potential of 17.5 ± 3.5 mV, and an MB loading efficiency of 81.2 ± 3.5%. **Step 4‐2** portrays the detection of a specific membrane protein (mProtein) of interest on the captured EV subpopulation, achieved through fluorescence‐labeled detection antibody probes. High‐resolution total internal reflection fluorescence (TIRF) microscopy was employed to record one hundred fluorescence images in a single well. **Step 5** displays a representative TIRF fluorescence image of the captured EVs with probes detecting biomarkers on the biochip surface. Subsequently, fluorescence image analysis was performed through a computer algorithm that generates a fluorescence intensity histogram of the bright spots detected in the TIRF images. A suitable histogram cutoff was established to minimize the influence of background noises and to emphasize the EVs containing high copies of mRNA and mProtein (i.e., the brighter spots in the image), as these EVs provided a more robust indication of the presence of cancer. The total fluorescence intensity (TFI) was determined as the sum of the histogram, that is, the area under the curve, after applying the cutoff. We examined the impact of non‐specific binding on exosomal CD63 protein expression using a mIgG isotype as a control. The findings indicate a significant increase in exosomal CD63 protein expression when captured with a CD63/CD9/CD81 antibody mixture, compared to the non‐specific mIgG capture (Figure S2B, Supporting Information). The TIRF images shown in Figure S2C, Supporting Information, further depict minimal non‐specific binding on the ILN biochips, when comparing samples with and without patient EVs.

The validity of MB applicability was first confirmed using two distinct MB designs that target separate local sequences within the GPC1 mRNA: one for 2052–2069 (NM_002081.3) and the other for 1173–1195 (NM_002081.3). Both MBs demonstrated similar GPC1 mRNA expression when tested across 10 healthy individuals and 10 PDAC patient samples (Figure 1B). For our study, we opted for NM_002081.3 whereby its effectiveness was validated through the serial dilution of synthetic standard vesicles. These vesicles comprised of anionic lipid nanoparticles containing GPC1 single‐stranded DNA (ssDNA) oligos, specifically the 18‐nucleotide sequence of 2052–2069. Notably, the fluorescence signal intensity exhibited a positive correlation with increased concentrations of the RNA oligos, with negligible signal alterations in the absence of the RNA oligos (Figure 1C).

EVs from cell‐conditioned medium and human plasma or serum samples can be purified by various methods, such as ultracentrifugation (UC), tangential flow filtration (TFF), size extrusion chromatography (SEC), and EV isolation kits.^[^ ^31^, ^32^ ^]^ Among them, UC with a sucrose gradient, SEC, and TFF can provide more refined EV samples with lower protein contamination than UC and EV isolation kits. Representative images of large and small EVs from a PDAC patient sample sorted by SEC (qEV column, 70 nm) were measured by transmission electron microscopy (TEM) (Figure 1D). However, SEC, UC with a sucrose gradient, and TFF are expensive and time‐consuming and thus not suitable for large‐scale clinical screening applications, where high‐throughput and low‐cost technologies are essential. Therefore, we isolated the EVs in liquid samples with commercially available EV isolation kits (Total Exosome Isolation (TEI) kit from Invitrogen). To reduce protein contamination for more refined EV samples, we added proteinase K (PK) during the EV isolation process. Through this adapted TEI‐PK EV isolation method, we successfully minimized inconsistencies in data associated with EV isolations. The EVs from PDAC patients (*n* = 20) exhibited an average particle size of 50–200 nm, with EV numbers ranging from 5E9 to 4E10 (Figure S2D, Supporting Information).

Through NanoSight nanoparticle tracking analysis, EVs sorted using the EV isolation kit from the conditioned media of PANC‐1 cells and the serum from an individual PDAC patient exhibited two prominent peaks with averaged diameters of 125 and 200 nm, respectively (Figure 1E). Further EV sorting was accomplished using the ILN biochip through an affinity‐based binding approach. In NanoSight nanoparticle tracking analysis, the recommended EV concentration ranged from 1E7 to 1E9 particles per mL. We selected a lower EV concentration range for the size distribution to better reveal the EV heterogeneity among different patient samples. As depicted in Figure S2E, Supporting Information, we noted distinct NTA curves between two PDAC patient samples at an EV concentration of E7 particles per mL. The Patient‐1 sample exhibited a single peak, whereas the Patient‐2 sample displayed two prominent peaks. The Patient‐2 sample was selected for representation in Figure 1E.

Distinct capture antibodies tethered on the surface of the ILN biochip were employed to target diverse EV subpopulations. Among the subpopulations, tetraspanin proteins like CD63, CD9, and CD81 were selected due to their prominent presence on the exosome surface.^[^ ^33^, ^34^, ^35^, ^36^ ^]^ Body fluids contain EVs originating from various cell types, with cancer cell‐derived EVs constituting only a small fraction, particularly during the early stages of the disease. Therefore, it was advantageous to capture cancer‐related EVs to enhance detection accuracy. Recent proteome analysis and profiling investigations have identified several highly expressed proteins on both PDAC cells and their secreted EVs. This list includes epithelial cell adhesion molecule (EpCAM), epidermal growth factor receptor (EGFR), human epidermal growth factor receptor‐2 (HER2), epithelial mucin 1 (MUC1), GPC1, Wnt family member‐2 (WNT2) and Glucose‐related protein‐94 (GRP94).^[^ ^15^ ^]^ We selected EpCAM, EGFR, and GPC1 antibodies for the capture of PDAC tMVs, because EpCAM and EGFR are the top two identified proteins^[^ ^15^ ^]^ and GPC1 is the protein of interest in this study.

In PANC‐1 cell‐derived EVs, GPC1 mRNA expression was found to be higher when captured with CD63, CD9, or CD81 antibodies, whereas the expression was lower with EpCAM, EGFR, or GPC1 antibodies (Figure 1F). In contrast, GPC1 mProtein expression was higher when captured with EpCAM, EGFR, or GPC1 antibodies, but lower with CD63, CD9, or CD81 antibodies (Figure 1G). These findings suggest variations in GPC1 mRNA and mProtein expression levels between EV subpopulations dominated by exosomes (captured using CD63, CD81, and CD9 antibodies) and tumor‐associated EVs (captured using EGFR, EpCAM, or GPC1 antibodies). To maximize efficacy, an antibody mixture of CD63/CD9/CD81 and EpCAM/EGFR/GPC1 in equal proportions was used in the subsequent investigations. The mixture demonstrated an enhanced effectiveness for GPC1 mRNA and GPC1 mProtein expression in both PANC‐1 cell‐derived EVs and EVs obtained from PDAC patients (Figure S3A,B, Supporting Information).

To calibrate and quantitatively analyze the molecular probes utilized in the ILN assay, we spiked varying dilutions of EVs derived from PANC‐1 cells into 1 mL of serum obtained from healthy donors (HD). This process enabled us to assess the linearity and limit of detection (LOD) of the assay. Our findings demonstrated a robust linear range with a LOD of 6.4E5 and 6.5E6 for GPC1 mRNA and mProtein detection in PANC‐1 EVs, respectively (Figure 1H,I). The qRT‐PCR and ELISA measurements for the average expression of EV GPC1 mRNA and mProtein, respectively, are presented in Figure 1H,I, showcasing a sensitivity enhancement of ≈100‐fold with our ILN biochip assay versus standard methods.

### 2.2 GPC1 mRNA and mProtein Expression in Distinct EV Subpopulations from Multiple PDAC Cell Lines and Human Serum

**Figure 2:**
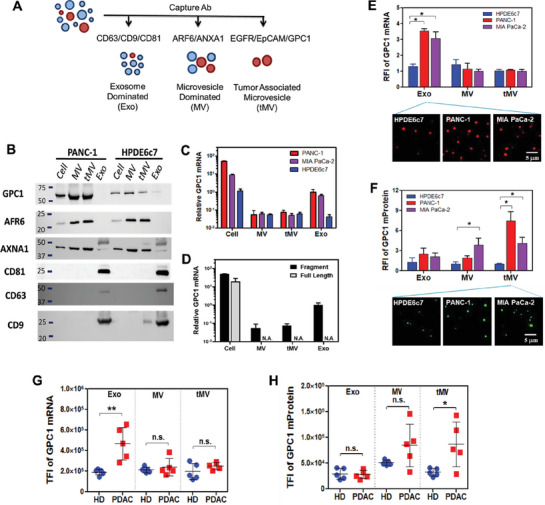
Distribution of GPC1 mRNA and protein in different EV subpopulations of non‐cancer and PDAC cells and human serum. A) Three sets of antibodies were used for capturing different EV subpopulations, including i) anti‐CD63/CD81/CD9 for exosome‐dominated vesicles (Exo), ii) anti‐ARF6/ANXA1 for microvesicle‐dominated vesicles (MV), and iii) anti‐EGFR/EpCAM/GPC1 for tumor‐associated microvesicle (tMV). B) Cell and vesicular GPC1 protein expression in each subpopulation (Exo, MV, and tMV) was measured by Western blot. Vesicular GPC1 protein is highly expressed in MV and tMV from the PANC‐1 cell line, but low in Exo. Lower GPC1 protein expression in a non‐cancerous cell line (HPDE6c7) and its EVs. C) GPC1 mRNA expression in cell, MV, tMV, and Exo were measured by qRT‐PCR. PANC‐1 and MIA PaCa‐2 cells expressed higher GPC1 mRNA expression in Exo but less in MV and tMV. D) By comparing qRT‐PCR results between two priming methods, random hexamer (Black, fragment) versus oligo dT (Gray, full length), we observed mostly GPC1 mRNA fragments rather than full length in Exo from PANC‐1 and MIA PaCa‐2 cells. Data were presented as means ± SD (*n* = 3). E) GPC1 mRNA expression in Exo, MV, and tMV in HPDE6c7, PANC‐1, and MIA PaCa‐2 cell‐derived EVs using the ILN biochip assay. F) GPC1 mProtein expression in Exo, MV, and tMV in HPDE6c7, PANC‐1, and MIA PaCa‐2 cell‐derived EVs using the ILN biochip assay. TIRF microscopy images were enlarged from 80 µm × 80 µm to 20 µm × 20 µm to show bright spots. RFI: Relative Fluorescence Intensity. G) GPC1 mRNA and H) GPC1 mProtein expression in Exo, MV, and tMV in HD and PDAC patient samples (*n* = 5). Data were presented as means (*n* = 2 wells, each well with 100 images). *p* values were determined by the paired two‐tailed Student's *t‐*test. *p < 0.05, **p < 0.01, n.s., not significant.

Concerns related to off‐target binding of molecular beacons and non‐specific binding of fluorescence‐labeled antibodies exist within the ILN biochip assay. To tackle this challenge, we collected EVs from distinct sources: a non‐cancer human pancreatic duct epithelial cell line (HPDE6c7) and two human PDAC cell lines (PANC‐1 and MIA PaCa‐2). We then conducted a comparison of GPC1 mRNA and protein expression measured by qPCR and Western blot against the ILN assay. The EVs were sorted into an exosome‐dominated subpopulation (Exo) using a 1/1/1 by weight mixture of CD63/CD81/CD9 antibodies, a microvesicle‐dominated subpopulation (MV) using a 1/1 by weight mixture of ARF6/Annexin A1 (ANXA1) antibodies,^[^ ^37^, ^38^ ^]^ and a PDAC tumor‐associated EV subpopulation (tEV) using a 1/1/1 by weight mixture of EGFR/EpCAM/GPC1 antibodies (**Figure** **2**A). A total of 30 ng proteins and 30–50 pg RNAs from cells or cell‐secreted EVs in culture medium were used in Western blot and qPCR measurements, respectively. The Western blot results revealed that the Exo subpopulation displayed high CD63, CD81, and CD9 expression, along with low ARF6 and ANXA1 expression. On the other hand, the MV subpopulation showed high ARF6 and ANXA1 expression, along with low CD63, CD81, and CD9 expression in both PANC‐1 and HPDE6c7 cell‐derived EVs (Figure 2B). Similar to the MV, tEV also demonstrated high ARF6 and ANXA1 expression, along with low CD63, CD81, and CD9 expression in both PANC‐1 and HPDE6c7 cell‐derived EVs. Therefore, we classified tEV as tumor‐associated microvesicle (tMV). Both MV and tMV derived from PANC‐1 cells showed a higher GPC1 protein expression than HPDE6c7 cells (Figure 2B). This trend was also observed in another PDAC cell line, MIA PaCa‐2 (Figure S4, Supporting Information). Conversely, qRT‐PCR results indicated that GPC1 mRNA expression was higher in the Exo subpopulation than in MVs and tMVs, evident in both PANC‐1 and MIA PaCa‐2 cell‐derived EVs compared to HPDE6c7 cell‐derived EVs (Figure 2C). Notably, GPC1 mRNA in EVs is primarily comprised of fragments, rather than full‐length mRNA, as observed in cells (Figure 2D).

Subsequently, we employed the ILN biochip assay to capture Exo, MV, and tMV subpopulations through antibody interactions and evaluated their GPC1 mRNA and mProtein expression at the individual EV level. In line with the qRT‐PCR findings, Exos derived from both PANC‐1 and MIA PaCa‐2 cells exhibited elevated GPC1 mRNA expression compared to those from HPDE6c7 cells. However, this was not the case in MVs and tMVs (Figure 2E). Regarding GPC1 mProtein expression, the ILN assay outcomes paralleled the Western blot results, demonstrating that both tMVs and MVs originating from PANC‐1 and MIA PaCa‐2 cells showed higher GPC1 mProtein expression than those from HPDE6c7 cells (Figure 2F).

We proceeded to assess the various EV subpopulations within serum samples collected from 5 healthy donors (HD; Zen‐bio, Inc.) and 5 PDAC patients (The Ohio State University; OSU) using the same antibody mixtures in the ILN biochip assay. Once again, GPC1 mRNA expression in the Exo subpopulation exhibited significantly higher levels in PDAC patients compared to HD samples. However, no notable differences were observed in MVs and tMVs (Figure 2G). Conversely, EV GPC1 mProtein expression remained low in Exos for both PDAC patients and HD samples. Interestingly, the MV subpopulation, particularly tMVs, was capable of effectively discriminating between PDAC and HD (Figure 2H). Collectively, the findings from both cell lines and human serum affirm the minimal off‐targeting effects of our molecular probes. The meticulous selection of capture antibodies emerges as a crucial factor in effectively sorting Exo, MV, or tMV subpopulations on the ILN biochip surface. This precise sorting is essential for capturing specific EV subpopulations for the detection of mRNA and mProtein biomarkers. Notably, GPC1 mProtein on the MV surface, particularly tMVs, and GPC1 mRNA in Exos demonstrated the capability to discern between cancer cells and non‐cancer cells or between PDAC patients and healthy donors with significant accuracy.

**Figure 3:**
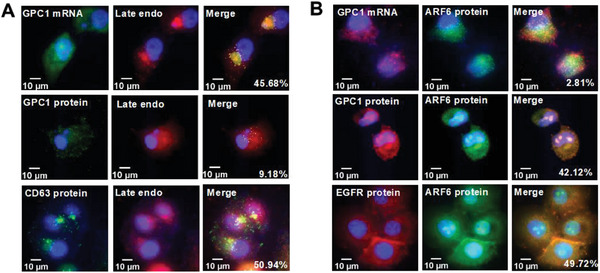
GPC1 mRNA and protein co‐localization with exosome and microvesicle markers. A) Co‐localization images of GPC1 mRNA, GPC1 protein, and CD63 protein with late endosome (endo)/ILV marker Rab7 in PANC‐1 cells. B) Co‐localization images of GPC1 mRNA, GPC1 protein, and EGFR protein with MV marker ARF6 in PANC‐1 cells.

Given that EVs are released from cells, our subsequent investigation delved into the distribution of GPC1 mRNA and protein within selected cellular compartments in PANC‐1 cells through immunofluorescence. Approximately half of the GPC1 mRNA, but only <10% of the GPC1 protein, exhibited co‐localization in late endosomes by co‐staining the Rab7 protein, a well‐established late endosome marker (**Figure** **3**A). Notably, the CD63 protein, a recognized marker for multivesicular body (MVB)/intraluminal vesicle (ILV), exhibited a strong co‐localization within late endosomes (Figure 3A). This co‐localization pattern aligns with EV biogenesis,^[^ ^39^ ^]^ wherein Exos originate from ILVs within MVBs in the cell cytoplasm.^[^ ^40^, ^41^ ^]^ Conversely, the GPC1 protein, distinct from its mRNA counterpart, displayed a substantial degree of co‐localization with the MV marker ARF6 (Figure 3B). Further supporting this, other MV markers like CD40 and selectin also exhibited high levels of co‐localization with the GPC1 protein (Figure S5, Supporting Information). Certain membrane proteins, such as epidermal growth factor receptor (EGFR)^[^ ^42^ ^]^ were also integrated into MVs that were secreted from the cell surface (Figure 3B).

GPC1 overexpression on the plasma membrane binds growth factors, cytokines, and other factors via its matured heparin sulfate side chains.^[^ ^43^ ^]^ The uptake and stimulation of these factors strongly induce the recycling of GPC1 via endocytosis.^[^ ^44^, ^45^ ^]^ As the GPC1 protein enters the realm of intracellular vesicular trafficking for recycling, it becomes susceptible to swift cleavage and subsequent degradation by endosomal heparinase. This enzyme is notably overexpressed in all aggressive and metastatic cancers.^[^ ^46^ ^]^ Enzyme activity leads to the rapid breakdown of the full structure of the N‐glycosylated GPC1 protein. As a result, the full GPC1 protein structure is far less prevalent within EVs formed from intracellular vesicles such as Exos. This phenomenon elucidates the rationale behind the prominence of the GPC1 mProtein on the MV or tMV surface, instead of in Exos. Careful selection of the appropriate EV subpopulations is imperative for the effective detection of the GPC1 protein and its corresponding mRNA as a cancer biomarker for PDAC.

### 2.3 GPC1 Exo‐mRNA and tMV‐mProtein Expression as a Biomarker for Clinical PDAC Diagnosis

**Figure 4:**
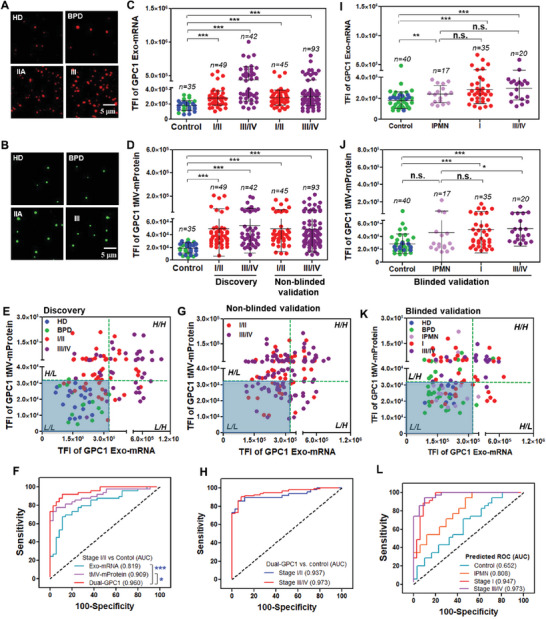
The ILN biochip assay of GPC1 Exo‐mRNA and tMV‐mProtein expression for discovery, non‐blinded validation, and blinded validation studies. A,B) Representative TIRF images for GPC1 Exo‐mRNA and tMV‐mProtein expression. C) GPC1 Exo‐mRNA expression for discovery and non‐blinded validation studies. D) GPC1 tMV‐mProtein expression for discovery and non‐blinded validation studies. TIRF images were enlarged from 80 µm × 80 µm to 20 µm × 20 µm to show bright spots. E) Scatter plot for discovery samples from OSU. F) ROC curves of GPC1 Exo‐mRNA and tMV‐mProtein expression as a single‐ or dual‐marker for Stage I/II PDAC patient for discovery set compared to control. G) Scatter plot for non‐blinded validation samples from combined MSKCC and TVGH. H) ROC curves of dual GPC1 Exo‐mRNA and tMV‐mProtein expression for Stage I/II and Stage III/IV PDAC patients for non‐blinded validation set compared to control. I,J) GPC1 Exo‐mRNA and tMV‐mProtein expression for blinded validation samples. K) Scatter plot for blinded validation samples. L) Predicted ROC curves for dual‐GPC1 expression for blinded validation samples. Pairwise comparison *p* values were determined by the Mann–Whitney U test. *p < 0.05, ***p < 0.001, n.s., not significant. Dotted lines in (E, G, and K) indicate the cut‐off values from the control (green). All data were presented as means (*n* = 2 wells, each well with 100 images).

We then proceeded to select GPC1 mRNA expression in Exos and GPC1 mProtein expression in tMVs to analyze additional serum samples from PDAC patients at different stages (*n* = 91) from OSU, along with HD (*n* = 20 from Zen‐bio, Inc.), and patients with BPD (*n* = 15 from Taipei Veterans General Hospital (TVGH) in Taiwan). Details regarding the clinical characteristics of these patients are provided in **Table** **1** and Table S1, Supporting Information. Due to the limited amount of Stage I samples, we combined Stage IA/IB, Stage IIA, and Stage IIB samples as a single cohort as tumors at these stages are considered “resectable.” Similarly, Stage III and Stage IV samples were grouped into a late‐stage “non‐resectable” cohort. Our ILN biochip assay unveiled notably higher TFI values for either GPC1 Exo‐mRNA or GPC1 tMV‐mProtein expression in both Stage I/II and Stage III/IV PDAC patients compared to HD and BPD (***p < 0.001 for both GPC1 tMV‐mProtein and GPC1 Exo‐mRNA; **Figure** **4**A–D). There were no discernible differences in GPC1 Exo‐mRNA or GPC1 tMV‐mProtein expression levels between HD and BPD samples. Consequently, we compared EV GPC1 expression in PDAC patients by merging HD and BPD samples as a single control cohort in the remaining study.

**Table 1:** Clinical characteristics of PDAC patients.

Characteristics	PDAC samples (Biomarker screening)					PDAC samples (Chemotherapy prognosis)		
	Discovery	Non‐blinded validation		Blinded validation				
Hospitals	OSU (n = 91)	MSKCC (n = 65)	TVGH (n = 73)	TVGH (n = 40)	NCKUH (n = 15)	CGMH (n = 22)	TVGH (n = 40)	NCKUH (n = 44)
Age (years)								
Median (range)	69 (37‐88)	65 (35‐89)	66 (42‐84)	65 (47‐84)	75 (41‐80)	63.5 (41‐81)	68 (40‐84)	64 (32‐88)
Gender, n (%)								
Male	43 (47.3%)	41 (63.1%)	46 (53.3%)	21 (52.5%)	6 (40.0%)	12 (54.5%)	30 (75.0%)	22 (50%)
Female	48 (52.7%)	24 (36.9%)	27 (46.7%)	19 (47.5%)	9 (60.0%)	10 (45.5%)	10 (25.0%)	22 (50%)
Stage at diagnosis, n( %)								
IA/IB	6 (6.6%)	3 (4.6%)	25 (34.2%)	20 (50.0%)	15 (100%)	–	1 (2.5%)	2 (4.5%)
IIA/IIB	43 (47.2%)	11 (16.9%)	16 (21.9%)	–	–	–	–	1 (2.3%)
III	30 (33.0%)	12 (18.5%)	6 (0.08%)	10 (25.0%)	–	6 (27.3%)	3 (7.5%)	8 (18.2%)
IV	12 (13.2%)	39 (60.0%)	36 (49.3%)	10 (25.0%)	–	16 (63.7%)	36 (90.0%)	33 (75.0%)
CA19‐9 levels								
Low (≤ 37)	ND	15 (23.1%)	19 (26.0%)	12 (30.0%)	5 (33.3%)	8 (36.4%)	10 (25.0%)	6 (13.6%)
High (>37)	ND	50 (76.9%)	54 (74.0%)	28 (70.0%)	10 (66.7%)	14 (63.7%)	30 (75.0%)	38 (86.4%)

ND: not determined.

Considering a substantial overlap in EV GPC1 expression between PDAC patients and controls, we assessed a combined analysis of GPC1 Exo‐mRNA and tMV‐mProtein expression as a dual‐biomarker for PDAC detection. The scatter plot depicted in Figure 4E illustrates that all control samples exhibited low levels of Exo‐mRNA and tMV‐mProtein expression of GPC1 (referred to as L/L) in serum. On the other hand, PDAC patients revealed either high Exo‐mRNA and high tMV‐mProtein (H/H), high Exo‐mRNA and low tMV‐mProtein (H/L), or low Exo‐mRNA and high tMV‐mProtein (L/H) expression of GPC1 in serum. In terms of diagnostic performance, the area under the curve (AUC) in the receiver operating characteristic (ROC) analysis for patients with Stage I/II PDAC was 0.819, 0.909, and 0.960 for GPC1 Exo‐mRNA, tMV‐mProtein, and the GPC1 Exo‐mRNA/tMV‐mProtein (dual‐GPC1), respectively, compared to the control (Figure 4F). The dual‐GPC1 marker outperformed the individual Exo‐mRNA (***p < 0.001) or tMV‐mProtein marker (*p < 0.05). Similar findings were also evident for PDAC patients with Stage III/IV (Figure S6, Supporting Information). To assess significant differences between cohorts, we utilized the central limit theorem to compute the minimum sample size required for a practical normal distribution. In the case of the discovery set, we obtained a power of 0.999 and 1.0 for Stage I/II (*n* = 42) and Stage III/IV (*n* = 49) versus the control (HD and BPD) (*n* = 35), respectively. A power greater than 0.8 suggests that the sample size is statistically significant for discerning differences between cohorts.

To distinguish PDAC patients from the controls in a clinical setting, distinct optical cut‐off values from TIRF images were established for Exo‐mRNA (TFI = 325,873) and tMV‐mProtein (TFI = 32,495), determined with 100% specificity from the ROC curves of the discovery cohort. These optical cut‐off values can be translated into analytical quantities, such as 6.5E7 and 2.0E8 PANC‐1 EVs per mL in HD serum for GPC1 Exo‐mRNA and tMV‐mProtein, respectively, based on the calibration curves illustrated in Figure 1H,I. The calibrated analytical cut‐off values thus enable the translation of the assay for reproducible outcomes attained from various hospitals/laboratories that employ distinct TIRF microscopes.

### 2.4 Validation of GPC1 Exo‐mRNA and tMV‐mProtein Expression in PDAC Diagnosis

We next conducted non‐blinded and blinded validation studies involving 193 clinical PDAC patient samples obtained from three distinct hospitals located in the US and Taiwan. The clinical characteristics of these samples are likewise detailed in Table 1.

#### 2.4.1 Non‐Blinded Validation

Utilizing the cut‐off values determined from the discovery set (OSU), we assessed PDAC samples from the Memorial Sloan Kettering Cancer Center (MSKCC) in New York City (*n* = 65) and TVGH (*n* = 73) in Taiwan. Given the limited number of patient samples in each hospital, we merged the samples from both institutes. The GPC1 dot charts and scatter plots for patients from each hospital are presented in Figure S7, Supporting Information. Our ILN biochip assay demonstrated that both GPC1 Exo‐mRNA (Figure 4C) and tMV‐mProtein (Figure 4D) expression exhibited significantly higher levels in PDAC patients with Stage I/II (**p < 0.01 for both) and Stage III/IV (***p < 0.001 for both) from both hospitals, in comparison to the control cohort. The dual‐GPC1 Exo‐mRNA/tMV‐mProtein (Figure 4G) effectively discriminated PDAC patients from the control cohort, with AUC/ROC values of 0.937 and 0.973 for Stage I/II and Stage III/IV, respectively (Figure 4H).

#### 2.4.2 Blinded Validation

Subsequently, we conducted a blinded validation study utilizing a mixture of clinical patient and non‐patient plasma samples from TVGH. This included Stage I PDAC (*n* = 20), Stage III/IV PDAC (*n* = 20), IPMN (intraductal papillary mucinous neoplasm, *n* = 17), BPD (*n* = 6), and HD (*n* = 10). Additionally, plasma samples from National Cheng Kung University Hospital (NCKUH) were included, consisting of BPD (*n* = 24) and Stage I PDAC (*n* = 15). Similar to the non‐blinded validation, PDAC patient samples from TVGH and NCKUH were combined. The GPC1 dot charts and scatter plots for the patients from each hospital are presented in Figure S8, Supporting Information. Both Stage I and Stage III/IV PDAC patients exhibited significantly elevated GPC1 Exo‐mRNA and tMV‐mProtein expression compared to control of HD and BPD (***p < 0.001) (Figure 4I,K). By employing the cut‐off derived from Figure 4C, we achieved high AUC/ROC values of 0.947 for Stage I and 0.973 for Stage III/IV PDAC (Figure 4L). The GPC1 Exo‐mRNA/tMV‐mProtein expression demonstrated some differentiation between IPMN and the control group, with an AUC/ROC of 0.808 (Figure 4L), likely due to the presence of pancreatic cancer precursor lesions in some IPMN patients.^[^ ^24^ ^]^ Conversely, a few BPD cases exhibited slightly elevated GPC1 Exo‐mRNA or tMV‐mProtein expression, yielding an AUC/ROC of 0.652 compared to the control group (Figure 4L). For the non‐blinded validation set, we achieved a power close to 1.0 for both Stage I/II (*n* = 45) and Stage III/IV (*n* = 93) versus the control (HD and BPD) (*n* = 35). For the blinded validation set, we achieved a power of 0.916 and 0. 985 for Stage I/II (*n* = 35) and Stage III/IV (*n* = 20) versus the control (HD and BPD) (*n* = 40), respectively.

### 2.5 GPC1 Exo‐mRNA/tMV‐mProtein Superior to CA19‐9 Expression in PDAC Diagnosis

**Figure 5:**
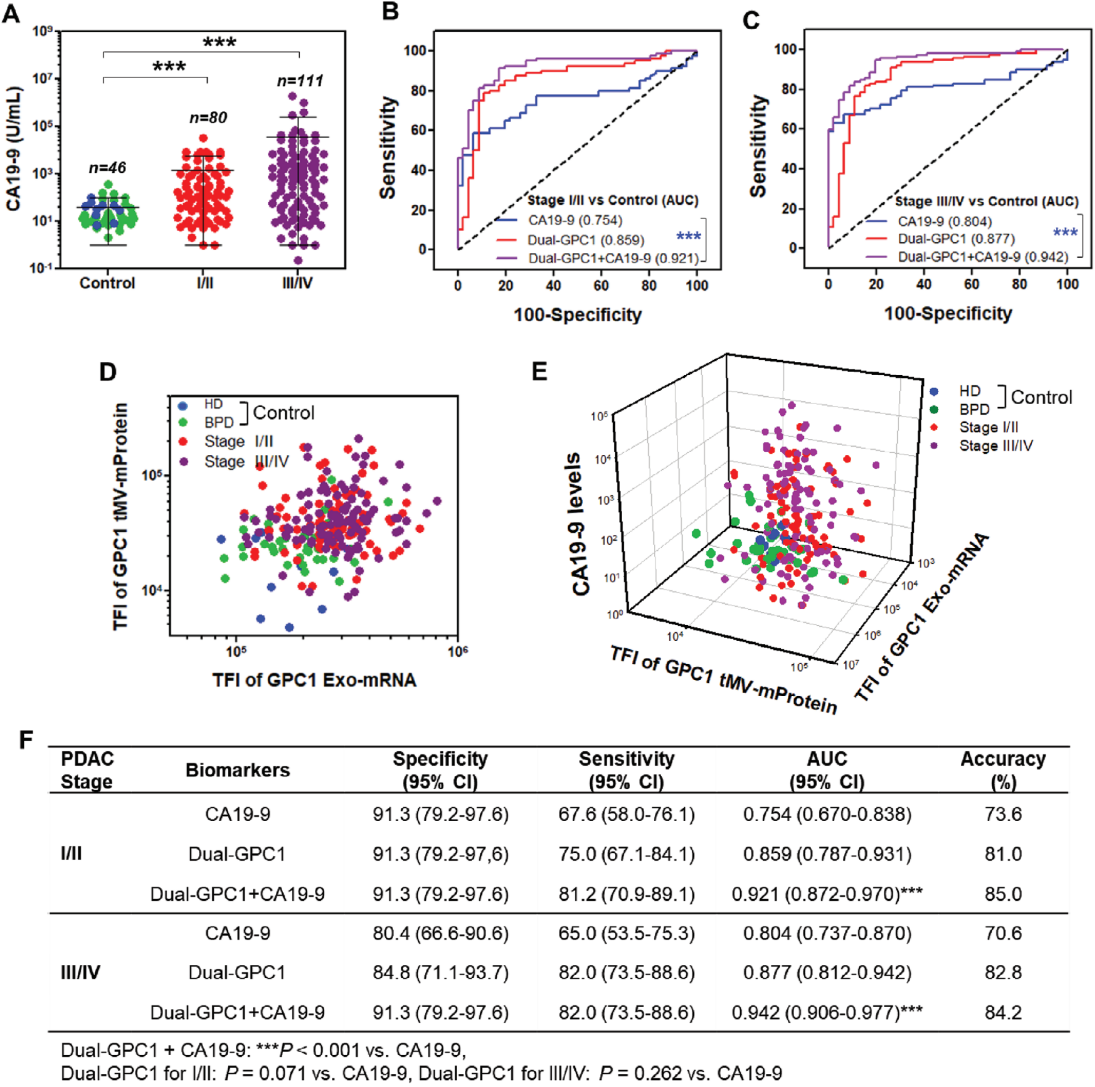
Blood CA19‐9 levels and GPC1 Exo‐mRNA/tMV‐mProtein expression as single‐, dual‐ or triple‐biomarkers for PDAC patients with Stage I/II and Stage III/IV. A) CA19‐9 levels in patients with Stage I/II and III/IV. B,C) ROC curves for PDAC patients with Stage I/II and III/IV compared to the control. D) Scatter plot of GPC1 Exo‐mRNA and tMV‐mProtein expression for patients with Stage I/II and III/IV. E) 3D scatter plot of CA19‐9, GPC1 Exo‐mRNA, and GPC1 tMV‐mProtein for PDAC patients with Stage I/II and III/IV. F) Statistical analysis of ROC curves of CA19‐9 and GPC1 Exo‐mRNA/tMV‐mProtein as single‐, dual‐ or triple biomarkers. Pairwise comparison *p* values were determined by the Mann–Whitney U test. ***p < 0.001. All data were presented as means (*n* = 2 wells, each well with 100 images).

Level of CA19‐9 in blood serves as the established clinical standard biomarker for PDAC patients. Within the MSKCC samples, a subset exhibited notably low CA19‐9 expression. EV GPC1 expression as determined by our ILN biochip assay, effectively identified these PDAC patients (Figure S9, Supporting Information). Consequently, we compared CA19‐9 levels against dual‐GPC1 Exo‐mRNA/tMV‐mProtein expression in single EVs using the dataset of all 193 PDAC patient samples in both non‐blinded and blinded validation studies. Blood CA19‐9 levels were notably elevated in PDAC patients with Stage I/II (***p < 0.001) and Stage III/IV (***p < 0.001) in comparison to the control group (**Figure** **5**A), yielding AUC/ROC values of 0.754 and 0.804, respectively (Figure 5B,C), which align with previously reported AUC/ROC values for CA19‐9 levels.^[^ ^24^ ^]^ In comparison, dual‐GPC1 Exo‐mRNA/tMV‐mProtein expression in a scatter plot (Figure 5D), with AUC/ROC values of 0.859 and 0.877 for Stage I/II and III/IV, respectively (Figure 5B,C), demonstrated better diagnostic performance. Evidently, dual‐GPC1 Exo‐mRNA/tMV‐mProtein expression, as determined by our ILN biochip assay, surpassed CA19‐9 levels in PDAC diagnosis. Furthermore, combining CA19‐9 levels in blood^[^ ^47^, ^48^ ^]^ and dual‐GPC1 Exo‐mRNA/tMV‐mProtein expression (Figure 5E) led to a substantial enhancement in diagnostic accuracy, resulting in AUC/ROC values of 0.921 and 0.947 for Stage I/II and Stage III/IV PDAC patients, respectively (Figure 5B,C). An AUC in the range of 0.7–0.8 as observed with CA19‐9 is deemed acceptable in diagnostic test assessments, 0.8–0.9 as observed with dual‐GPC1 Exo‐mRNA/tMV‐mProtein is classified as excellent, and values exceeding 0.9 as observed by combining Exo‐mRNA/tMV‐mProtein with CA19‐9 are considered outstanding.^[^ ^49^ ^]^ A more comprehensive analysis is given in Figure 5F where the combined CA19‐9 and EV dual‐GPC1 expression statistically outperformed CA19‐9 alone for both Stage I/II and Stage III/IV patients (***p < 0.001).

### 2.6 GPC1 Exo‐mRNA/tMV‐mProtein Expression in Other Cancers

**Figure 6:**
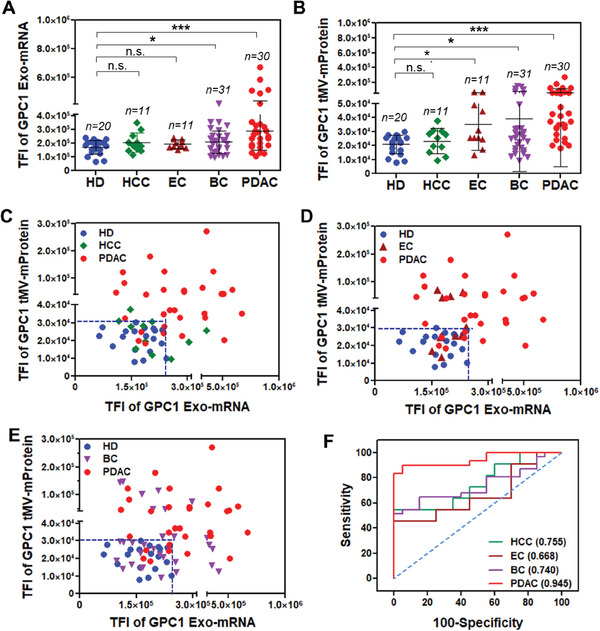
GPC1 Exo‐mRNA/tMV‐mProtein expression for PDAC and non‐PDAC cancer patient samples. GPC1 A) Exo‐mRNA and B) tMV‐mProtein expression in patients with HCC, EC, BC, and PDAC. Scatter plot of GPC1 Exo‐mRNA and tMV‐mProtein expression in patients with C) HCC, D) EC, and E) BC. F) ROC curves of GPC1 Exo‐mRNA/tMV‐mProtein expression for PDAC, BC, HCC, and EC patients versus HD. HCC: hepatocellular carcinoma; EC: esophageal cancer; BC: breast cancer; Pairwise comparison *p* values were determined by the Mann–Whitney U test. *p < 0.05, **p < 0.01, ***p < 0.001, n.s. not significant. All data were presented as means (*n* = 2 wells, each well with 100 images).

Similar to CA19‐9, GPC1 upregulation has also been observed in other cancer types.^[^ ^17^, ^18^, ^19^, ^20^, ^21^, ^22^, ^23^ ^]^ Therefore, we measured the expression of GPC1 Exo‐mRNA and tMV‐mProtein in serum samples from late‐stage breast cancer (BC) patients (MSKCC, *n* = 31), plasma samples from late‐stage hepatocellular carcinoma (HCC) patients (National Health Research Institute (NHRI) Biobank, *n* = 11), and plasma samples from late‐stage esophageal cancer (EC) patients (Chang Guan Memorial Hospital (CGMH), *n* = 11). We used plasma samples from Stage I PDAC patients from TVGH (*n* = 30) as a basis for comparison. In contrast to the distinct findings in PDAC, patients with BC only revealed a weak statistical difference (*p < 0.05) in GPC1 Exo‐mRNA or tMV‐mProtein expression. Conversely, patients with HCC did not display notable differences in GPC1 Exo‐mRNA or tMV‐mProtein expression when compared to HD (*n* = 20 from Zen‐bio, Inc). For patients with EC, a significant difference was observed in GPC1 tMV‐mProtein, but not in Exo‐mRNA, compared to HD (**Figure** **6**A,B). When considering the combined GPC1 Exo‐mRNA/tMV‐mProtein expression as was performed for PDAC, the ILN biochip assay was not able to effectively discriminate patients with HCC (Figure 6C), EC (Figure 6D), and BC (Figure 6E) from healthy donors. The corresponding AUC/ROC values were 0.755, 0.668, and 0.740, respectively. In contrast, the AUC/ROC value of the PDAC samples was substantially higher at 0.945 (Figure 6F). Taken together, combined GPC1 Exo‐mRNA/tMV‐mProtein expression holds significant promise for PDAC screening with a limited specificity for other cancer types.

### 2.7 GPC1 tMV‐mProtein and Exo‐mRNA as a Prognosis Biomarker for PDAC Patients Undergoing Chemotherapy

**Figure 7:**
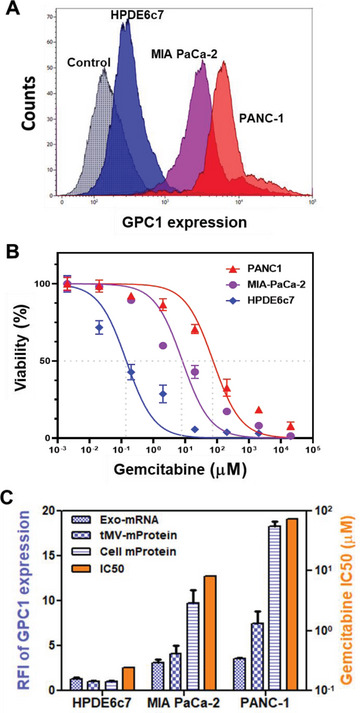
GPC1 expression and IC_50_ of Gemcitabine in pancreatic cancer cells and non‐cancer pancreatic cells. A) GPC1 protein expression in HPDE6c7, MIA PaCa‐2, and PANC‐1 cells by flow cytometry. B) Cell viability after 48 h treatment of Gemcitabine in HPDE6c7, MIA PaCa‐2 and PANC‐1 cells by MTS assay. C) Relative GPC1 Exo‐mRNA, tMV‐mProtein, and cell‐mProtein expression with IC_50_ of Gemcitabine in HPDE6c7, MIA PaCa‐2, and PANC‐1 cells and their EVs. Data were presented as means ± SD (*n* = 3).

It is recognized that elevated GPC1 expression is associated with an unfavorable prognosis in PDAC.^[^ ^50^ ^]^ Additionally, the expression of GPC1 protein on PDAC cell lines influences their resistance to chemotherapeutic drugs.^[^ ^51^, ^52^, ^53^ ^]^ To ascertain the levels of the GPC1 membrane protein expression in pancreatic cancer cells, we conducted flow cytometry (Figure **7**A). Furthermore, we measured the IC_50_ (half maximal inhibitory concentration) of the first‐line chemotherapeutic drug, Gemcitabine, using an MTS assay (Figure 7B). PANC‐1 cells with high GPC1 protein expression demonstrated enhanced resistance to Gemcitabine with an IC_50_ of 74.3 µm. Conversely, MIA‐PaCa‐2 cells displaying moderate GPC1 mProtein expression exhibited a lower resistance to Gemcitabine with an IC_50_ of 8.1 µm. In stark contrast, HPDE6c7 cells, characterized by lower GPC1 expression, displayed a heightened sensitivity to Gemcitabine, with an IC_50_ of 0.24 µm. Similar trends were also evident in terms of tMV‐mProtein expression and Exo‐mRNA expression (Figure 7C).

**Figure 8:**
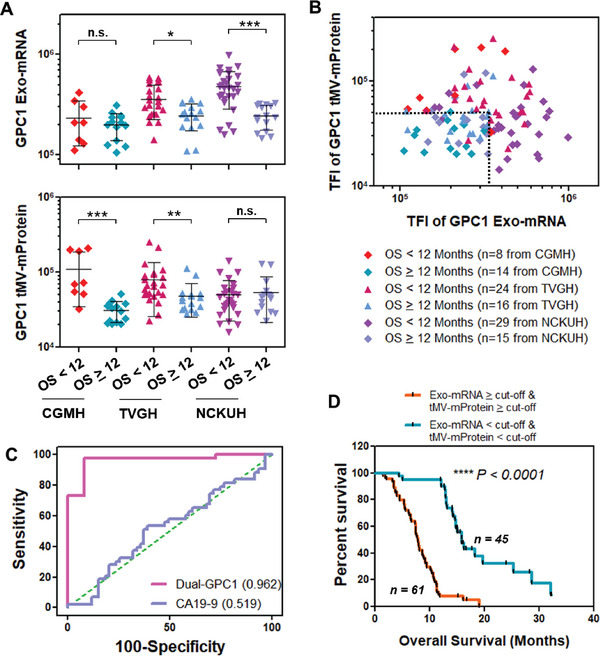
GPC1 Exo‐mRNA and tMV‐mProtein dual‐biomarker for pancreatic cancer prognosis in chemotherapy. A) GPC1 Exo‐mRNA and GPC1 tMV‐mProtein expression of mostly late‐stage PDAC patients from CGMH, TVGH, and NCKUH before chemotherapy (C0) with <12 months or ≥12 months overall survival (OS). B) Scatter plot of GPC1 Exo‐mRNA versus GPC1 tMV‐mProtein expression at C0. C) AUC/ROC of dual‐GPC1 and CA19‐9 with OS ≥12 months compared to OS <12 months. D) Kaplan‐Meier curves (log‐rank test) of OS based on cut‐off values of GPC1 Exo‐mRNA and tMV‐mProtein expression at C0. Cut‐off value for GPC1 Exo‐mRNA (TFI = 331,725 or 7E7 PANC‐1 EVs per mL HD serum) and mProtein (TFI = 51,480 or 6E8 PANC‐1 EVs per mL HD serum) were determined using AUC/ROC and calibration curves. Pairwise comparison *P* values were determined by the Mann–Whitney U test. *p < 0.05, **p < 0.01, ***p < 0.001, ****p < 0.0001, n.s. not significant. All data were presented as means (*n* = 2 wells, each well with 100 images).

We proceeded to investigate whether the expression of GPC1 Exo‐mRNA and tMV‐mProtein in blood could potentially function as prognostic biomarkers for PDAC patients undergoing chemotherapy. We collected a total of 106 patient serum samples before commencing chemotherapy (denoted as C0) from three hospitals in Taiwan: CGMH (*n* = 22), TVGH (*n* = 40), and NCKUH (*n* = 44). All patients had documented overall survival (OS) data, and the majorities were in the late stage of the disease. The patient clinical characteristics and chemotherapy regimens are summarized in Tables S2–S4, Supporting Information. When we divided the patients into two cohorts based on an OS threshold of 12 months, we did not observe any discernible trend using blood CA 19‐9 expression before treatment (C0) as a biomarker (Figure S10, Supporting Information). In contrast, the expression of GPC1 Exo‐mRNA and GPC1 tMV‐mProtein were notably lower in patients with an OS ≥12 months compared to those with an OS <12 months (Figure **8**A). Similar to PDAC screening, patients with longer OS had both low Exo‐mRNA and low tMV‐mProtein expression of GPC1 (i.e., L/L) in plasma. Conversely, patients with shorter OS exhibited varying combinations of Exo‐mRNA and high tMV‐mProtein expression (i.e., H/H, H/L, or L/H) in their plasma samples (Figure 8B). Employing a dual‐GPC1 Exo‐mRNA and tMV‐mProtein biomarker at C0, we achieved a remarkable ability to discriminate patients with OS ≥12 months from those with OS <12 months with an impressive AUC/ROC of 0.962 (Figure 8C). In contrast, the CA19‐9 levels in the blood at C0 displayed poor discrimination power, yielding an AUC/ROC of 0.519 for distinguishing between OS <12 months and OS ≥12 months. To establish the distinct cut‐off values of Exo‐mRNA (TFI = 331,725 or 7E7 PANC‐1 EVs per mL HD serum) and tMV‐mProtein (TFI = 51,480 or 6E8 PANC‐1 EVs per mL HD serum) for distinguishing patients with longer or shorter survival, we employed the AUC/ROC and calibration curves in Figure 1H and I. Notably, patients with higher cut‐off values were statistically associated with a shorter OS (log‐rank test, ****p < 0.0001) (Figure 8D).

## 3 Discussion

**Figure 9:**
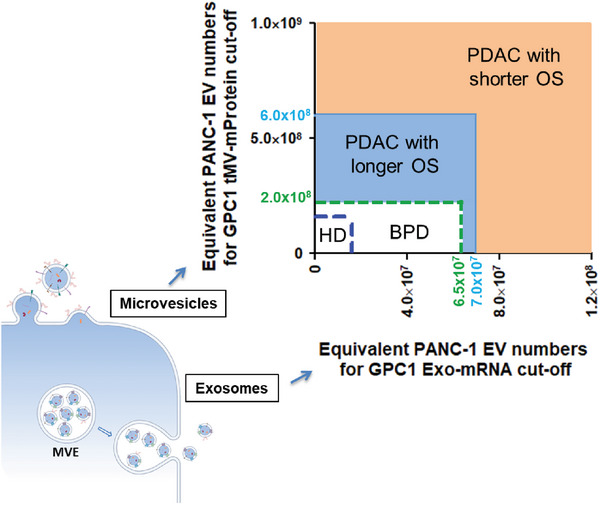
Dual GPC1 Exo‐mRNA and tMV‐mProtein biomarker for PDAC screening and prediction of prognostic outcome. Graphical summary showing that a combination of GPC1 mRNA expression in Exos and the GPC1 membrane protein expression in tMVs can serve as a viable biomarker for PDAC detection. The control group (HD and BPD) exhibited low GPC1 Exo‐mRNA expression (TFI ≤ 3.2E5 equivalent to 6.5E7 PANC‐1 EVs in 1 mL blood) and tMV‐mProtein expression (TFI ≤ 3.2E4 equivalent to 2.0E8 PANC‐1 EVs in 1 mL blood). PDAC patients with moderate GPC1 Exo‐mRNA expression (2.4E5 < TFI < 3.2E5 equivalent to ≈1.6E7–7.0E7 PANC‐1 EVs in 1 mL blood) and tMV‐mProtein expression (3.0E4 < TFI < 5.1E4 equivalent to ≈1.6E8–6.0E8 PANC‐1 EVs in 1 mL blood) were associated with longer OS. PDAC patients with high GPC1 Exo‐mRNA and tMV‐mProtein expression showed poor outcomes undergoing chemotherapy.

In this study, we have demonstrated that a single gene resulting from oncogenic activation, such as GPC1 is strongly associated with PDAC. By capturing and characterizing GPC1 mRNA expression within an exosome‐dominated EV subpopulation (Exo), as well as its membrane protein expression on tumor‐associated microvesicles (tMV), we have unveiled a potential single‐gene, dual‐biomarker strategy for the diagnosis and prognosis of PDAC. Leveraging a substantial number of clinical samples from multiple hospitals, we have showcased that the dual GPC1 tMV‐mProtein and Exo‐mRNA expression hold the potential to achieve good performance in early PDAC screening. By adding the conventional CA19‐9 protein marker to our Exo‐mRNA/tMV‐mProtein assay, the combined assay can reach exceptional sensitivity, specificity, and accuracy, in comparison to CA19‐9 alone. Moreover, the expression of GPC1 Exo‐mRNA and tMV‐mProtein demonstrates promise in PDAC prognosis for late‐stage patients undergoing chemotherapy (**Figure** **9**).

Although our experimental results of GPC1 mRNA and membrane protein expression in selected EV subpopulations perform better than other biomarkers reported for PDAC diagnosis and prognosis (see Table S5, Supporting Information), they are insufficient as a perfect biomarker for PDAC. The potential inclusion of additional coding genes and proteins resulting from oncogenic activation, alongside GPC1, could lead to the development of a more comprehensive PDAC biomarker classifier for both diagnosis and prognosis.

In our patient cohort, we observed that GPC1 tMV‐mProtein and Exo‐mRNA changes did not follow the same trend. A substantial number of patient samples exhibited a higher tMV‐mProtein expression but a lower Exo‐mRNA expression or vice versa. Since the expression of mRNAs and their corresponding proteins in cancer cells and tumor tissues do not consistently exhibit the same trend (i.e., both high or both low),^[^ ^50^, ^54^, ^55^, ^56^ ^]^ it is unsurprising that the EV mRNA and its corresponding protein expression may not consistently align across different patients. Hence, we believe that combining both mRNA and its protein expression in proper EV subpopulations yields a more robust biomarker compared to using individual biomarkers in EV‐based liquid biopsies. Given the heterogeneity of cancer and the cellular origins of EVs, the actual mechanism is still unclear. Further investigation is required to ascertain whether this is linked to the heterogeneity of PDAC, particularly in cases where certain samples display markedly high EV mRNA (or protein) expression but notably low EV protein (or mRNA) expression.

It is worth noting that the patient samples were sourced from various hospitals, each with its own distinct sample handling procedures and collection practices. Despite potential variations caused by sample handling and storage time, our assay consistently exhibited a strong performance across all samples. With the establishment of a standardized operating procedure, the assay's performance could be further improved. For future clinical applications, extensive validation on a larger scale is crucial to translate the findings in this study and the developed ILN biochip assay into a robust tool for early PDAC screening and prognosis.

## 4 Experimental Section

### Cell Lines, Antibodies, and Molecular Beacons

The PANC‐1 and MIA PaCa‐2 cell lines were purchased from the American Type Culture Collection. Cells were maintained in Dulbecco's modified Eagle's medium supplemented with 10% fetal bovine serum, 100 U mL^−1^ of penicillin, and 100 µg mL^−1^ of streptomycin at 37 °C in a 5% CO_2_/95% air humidified atmosphere. The human duct epithelial cell line (HPDE6c7) was purchased from Sigma‐Aldrich and maintained in Keratinocyte Serum Free Growth Medium (Sigma 131–500A) with 50 µg mL^−1^ bovine pituitary extract (Sigma P1476), 5 ng mL^−1^ EGF (Sigma GF316) and 100 U mL^−1^ of penicillin and 100 µg mL^−1^ of streptomycin at 37 °C in a 5% CO_2_/95% air humidified atmosphere. For capture antibodies, biotinylated anti‐CD63 antibody (ab1334331), biotinylated anti‐CD9 antibody (ab28094), and biotinylated anti‐CD81 antibody (ab239238) were purchased from Abcam (Cambridge, MA). An anti‐EpCAM antibody (MAB960, R&D system), recombinant chimeric EGFR monoclonal antibody (Cetuximab, Erbitux, ImClone LLC), anti‐GPC1 antibody (MAB45191, R&D systems), anti‐ARF6 antibody (NBP2‐41263, Novus Biologicals), and anti‐Annexin A1 (ANXA1) antibody (AF3770, R&D systems) were purchased and biotinylated. EZ‐Link Micro Sulfo‐NHS Biotinylation kit (#21925, ThermoFisher) was used for antibody biotinylation according to the manufacturer's instructions. Alexa Fluor 647 anti‐GPC1 antibody (ab237290, Abcam), anti‐ARF‐6 antibody‐Alexa Fluor 488 (sc‐7971 AF488, Santa Cruz Biotechnology), PE mouse anti‐human CD40 (555589, BD Pharmingen), FITC mouse anti‐p‐selectin (A51079, Beckman), anti‐ARF‐6 antibody‐Alexa Fluor 488 (sc‐7971 AF488, Santa Cruz Biotechnology), anti‐CD63 antibody‐Alexa Fluor 488 (sc‐5275 AF488, Santa Cruz Biotechnology) were purchased and used for EV protein detection. Molecular beacons for GPC1 mRNA detection was custom‐synthesized by Integrated DNA Technologies, Inc. (San Diego, California). The sequence for GPC1 MB for sequence 2052–2069 is 5′‐ +GCC +TGC /iCy3/+CC C+TG C+TC A+GA GCC AAC TGA GCA GGG /3BHQ_2/−3′, while that for GPC1 MB for sequence 1173–1195 is 5′‐  +GAC +ACT /iCy3/+CTC +CAC +ACC +CGA TGA TGG GTG TGG AG /3BHQ_2/−3′.

### Preparation of Synthetic Standard Vesicles

To develop a standard for MB calibration, anionic lipid nanoparticles containing GPC1 single‐stranded DNA (ssDNA) oligo (18 nucleotides of the 2052–2069 sequence of the GPC1 mRNA), termed synthetic standard vesicles. They have a similar membrane structure as the EVs with a mean diameter of 151.7 nm and a slightly negative surface charge (−10.9 mV). The anionic lipid nanoparticle formulation is DOPE/linoleic acid/ DMG‐PEG with a molar ratio of 50/48/2 in 5 mg lipid per mL ethanol. Briefly, 2 µL GPC1 ssDNA (30 µm) and 6.4 µL of scramble oligonucleotide (300 µm, 21‐oligonucleotide) were mixed with 27.6 µL di‐ionized water and then mixed with 24 µL lipid stock solution. Synthetic standard vesicles containing 100% of a scramble oligonucleotide were also prepared as a control for comparison. After 5 min of sonication, the mixture was injected into 540 µL PBS and sonicated for 5 min at room temperature. The solution was then dialyzed against PBS at room temperature overnight using a MWCO 20 000 Da dialysis device to remove residual free ssDNA and scramble oligonucleotide.

### Cell Staining and Co‐Localization Analysis

PANC‐1 cells were grown overnight on 35 mm confocal dishes and washed with PBS. First, cells were fixed in 4% formaldehyde solution in PBS for 10 min at room temperature and then permeabilized with 0.2% (v/v) Triton X‐100 (Sigma‐Aldrich) in PBS at room temperature. The cells were subsequently rinsed with PBS and incubated with Hoechst 33342 (1:2,000 dilution) for 10 min at room temperature. For mRNA staining, cells were incubated with blocking buffer (R37620, ThermoFisher) for 1 h at room temperature and then incubated with 1 µΜ GPC1 molecular beacons (MBs) for 1 h at 37 °C. After washing three times with PBS, cells were sequentially incubated with different colors of fluorescence‐labeled monoclonal antibodies (e.g., anti‐Rab7, anti‐GPC1, or anti‐ARF6) in 1% bovine serum albumin (BSA) solution after blocking with 5% BSA in PBS solution for 1 h at room temperature. After washing, cell fluorescence images were taken using TIRF microscopy. Cell co‐localization was further quantitated using the Imaris software (BITPLANE, Oxford Instruments, Zurich, Switzerland). First, a co‐localized area σ of *Ch1* (e.g., anti‐Rab7 as ILV/MVB marker or ARF6 as microvesicle marker) and *Ch2* (e.g., GPC1 mRNA or GPC1 protein probes) were generated and then further calculated the proportion by pixel analysis as:

(1) $$\%\;GPC1\;mRNA\;in\;\frac{ILV}{MVB}=\frac{Colocalization\;area\;}{Entire\;area\;from\;Ch1\;of\frac{ILV}{MVB}}$$

### Biochip Fabrication

A cleaned high‐precision glass coverslip (D263M Glass, 24 × 75 nm rectangle, 0.15 mm thickness, Schott AG, Germany) was first activated using a UV‐ozone cleaner for 15 min. Thin layers of 2 nm thick Ti and 10 nm thick Au were sequentially deposited using a Denton‐e‐beam evaporator (DV‐502A, Moorestown, NJ). The Au‐coated glass was immersed into a linker solution (1%) in ethanol (200 Proof, Fisher Scientific) overnight at room temperature. The linker solution composed of 1‐thiahexa(ethylene oxide) lipidic anchor molecule WC14 (20‐tetradecyloxy‐3,6,7,12,15,18, 22‐heptaoxahexa‐tricontane‐1‐thiol), a lateral spacer β‐mercaptoethanol (β‐ME), and biotin‐PEG‐SH (molar ratio = 15:83:2). The coverslip was then washed three times with ethanol and air‐dried. The treated glass coverslip was attached to a 64‐well chamber (Grace Bio‐Labs ProPlate multi‐well chamber, Sigma‐Aldrich) and washed thoroughly with deionized (DI) water. Next, 0.005% (w/v) neutravidin‐conjugated gold nanoparticles in PBS were applied into each well of biochip for 2 h at room temperature on a shaker (Titer Plate Shaker, Speed = 2). After rinsing six times with PBS, the biochip was incubated with a capture antibody mixture overnight at 4 °C. The antibody mixture included 50 µg mL^−1^ each of anti‐CD63/anti‐CD9/anti‐CD81 antibodies or anti‐ARF6/anti‐Annexin A1 antibodies or anti‐EpCAM/anti‐EGFR/anti‐GPC1 antibodies. Antibodies were biotinylated using an EZ‐Link Micro Sulfo‐NHS‐Biotinylation kit (ThermoFisher) before use. After the antibodies were tethered onto the nanogold surface, the biochip was washed six times with PBS, and then blocked with 5% (w/v) BSA in PBS for 1 h at room temperature before EV capture.

### EV Isolation from Cell Culture

Cells were grown for 48 h in DMEM medium supplemented with 2% EV‐depleted fetal bovine serum (A272081, ThermoFisher). The conditioned medium was collected in 50 mL tubes and centrifuged at 2,500×g for 30 min to remove cell debris. The supernatant was concentrated to 1 mL using an Amicon Ultra centrifugal unit (10 kDa MWCO, Fisher Scientific) at 2,500× g for 30 min. To purify EVs, 0.5 mL was loaded onto a size exclusion qEV_70_ column and started to collect fractions immediately using PBS as the elution buffer. 0.5 mL of each fraction was collected in 15 sequential fractions. Then, each fraction was concentrated to 100 µL using Amicon Ultra‐4 centrifugal filters with 10 kDa MWCO. EVs were quantified using the NanoSight nanoparticle tracking analysis. Protein concentration was determined by a BCA assay. Fractions were stored at −80 °C for subsequent GPC1 mRNA detection, GPC1 protein detection, and Cryo‐transmission electron microscopy (Cryo‐TEM).

### Human Serum/Plasma Samples

This study encompasses sizable clinical patient samples sourced from five hospital biobanks in the US and Taiwan (Table 1 and Tables S1–S4, Supporting Information). This diverse collection underscores the adaptability of our novel biomarker assay across various patient populations, even when considering the distinct blood sample collection methods and timeframes at each hospital. Furthermore, the methodology facilitates the acquisition of a greater number of early‐stage PDAC patient samples.

For the diagnostic study, a comprehensive set of 359 samples was utilized. This set encompassed patients with PDAC (*n* = 284), benign pancreatic diseases (*n* = 45), and healthy donors (*n* = 30) drawn from both the US and Taiwan. Among these, 91 PDAC patient serum samples were provided by the Ohio State University Cancer Hospital biobank, and were designated as the discovery set, with approval from the Institutional Review Boards (IRB) under OSU Protocol #2008C0093. Additionally, the study included 65 PDAC patient serum samples acquired from the Memorial Sloan Kettering Cancer Center (MSKCC) biobank in New York City, which constituted a non‐blinded validation subset under MSKCC Protocol #06‐107. Given the limited availability of Stage I PDAC patient samples at the two US hospitals, additional PDAC patient samples from Taiwan were also included. The sets consisted of 113 PDAC patient plasma samples from Taipei Veterans General Hospital (TVGH) in Taiwan. These samples were divided into a non‐blinded validation subset (*n* = 73) and a blinded validation subset (*n* = 40), with IRB approval under TVGH Protocol #2022‐07‐032BC. Additionally, the study incorporated 21 BPD patient plasma samples, comprising 14 chronic pancreatitis (CP) and 7 serous cystadenoma (SCA) cases from TVGH. These samples were likewise divided into non‐blinded (*n* = 15) and blinded (*n* = 6) validation subsets. Furthermore, the study encompassed 15 Stage I PDAC patients and 24 BPD (11 CP, 6 SCA, 4 autoimmune pancreatitis (AI) and 3 mucinous cystadenoma (MPC)) plasma samples from National Cheng Kung University Hospital (NCKUH) in Taiwan (NCKUH IRB Protocol #B‐ER‐109‐154; #A‐ER‐109‐355; #B‐ER‐111‐302) as part of the blinded validation subset.

For the prognosis study, we incorporated 101 patients with documented overall survival from three distinct hospitals in Taiwan. Among these, 22 PDAC patient plasma samples were from Chang Guan Memorial Hospital (CGMH) in Keelung (CGMH Protocol #201801058A3, #202002116B0C102, XPRPG2H0041‐5), 44 PDAC patient plasma samples were from TVGH (TVGH Protocol #2022‐07‐032BC) and an additional 44 PDAC patient plasma samples were from NCKUH (NCKUH IRB Protocol #B‐ER‐109‐154; #A‐ER‐109‐355; #B‐ER‐111‐302). This analysis encompassed the combined data set of all patient samples in this study.

Moreover, a separate set of non‐PDAC cancer samples was assembled to evaluate the potential of GPC1 Exo‐mRNA and tMV‐mProtein expression as biomarkers for cancers other than PDAC. Serum samples were acquired from late‐stage breast cancer patients (MSKCC, *n* = 31), while plasma samples were sourced from late‐stage hepatocellular carcinoma patients through the National Health Research Institute (NHRI) Biobank in Taiwan (*n* = 11), and from late‐stage esophageal cancer patients at CGMH (*n* = 11).

### Serum and Plasma Sample Collection and EV Isolation

All plasma samples were extracted from blood collected using BD vacutainer tubes containing K2‐EDTA. These serum and plasma samples were then stored at −80 °C or in liquid nitrogen until needed. The EVs from patient samples were isolated using a Total Exosome Isolation (TEI) Kit from Invitrogen, with slight modifications to the procedure. Briefly, 150 µL of serum/plasma was initially treated with 5 µl of proteinase K (PK) for 10 min at room temperature, followed by the addition of 50 µL of TEI solution to precipitate EVs. After centrifugation, the precipitate was reconstituted with 150 µL of PBS. EV number, particle size, zeta potential, and protein contamination were then measured. The size distribution and concentration of EVs were examined through NanoSight nanoparticle tracking analysis and dynamic light scattering. Protein contamination was examined by BCA protein assay.

### Biochip Calibration Using PANC‐1 EVs

To investigate the detection limit of our ILN biochip assay, PANC‐1 EVs were spiked into HD EVs at different concentrations ranging from 3.2E7 to 2E10 EVs per mL, while the healthy donor EVs were kept constant at 1E12 EVs per mL. After a fourfold dilution of each sample, the GPC1 mRNA level in healthy donor EVs spiked with PANC‐1 EVs were measured using our ILN biochips and compared with qRT‐PCR. Similarly, the GPC1 mProtein levels in healthy donor EVs spiked with PANC‐1 EVs were measured using our ILN biochips and compared with ELISA.

### EV mRNA Detection

First, cationic lipid nanoparticles encapsulated with the molecular beacon (CLN‐MB) were synthesized. 10 mg mL^−1^ lipids stock solution in ethanol was prepared using 1,2‐dioleoyl‐3‐tromethylammonium‐propane (DOTAP, Avanti Polar Lipids), 1,2‐dioleoyl‐sn‐glycero‐3‐phosphocholine (DOPC, Avanti Polar Lipids), cholesterol and 1,2‐distearoyl‐sn‐glycero‐3‐phosphoethanolamine‐N‐[biotinyl(polyethylene glycol)−2000] (DSPE‐PEG2000‐biotin, Avanti Polar Lipids) at a molar ratio of 50/33/15/2. To synthesize CLN‐MBs, 20 µL lipid stock solution was mixed with 30 µL MB solution, which contained 1 µL MB (100 µm), 6 µL of scramble oligonucleotide (300 µM, 21‐oligonucleotide), and 24 µL PBS. After 5 min of sonication, the mixture was injected into 450 µL PBS and further sonicated for 1 min at room temperature. The CLN‐MB was then dialyzed against PBS at room temperature for 2 h using an MWCO 20,000 Da dialysis device to remove residual free MB and scramble oligonucleotide. The particle size and zeta potential (ζ) of CLN‐MB were determined using dynamic light scattering and ZetaPALS from Brookhaven Instruments Corp. (Worcestershire, NY). Next, EVs from cell lines or healthy donor and patient serum/plasma were diluted four times with PBS before use. 20 µL of EVs was loaded to each well on the biochip and incubated for 2 h at room temperature on a shaker. After washing with PBS, 20 µL of CLN‐MBs was added to the wells and incubated for 1 h at 37 °C. Then, the biochip was washed with PBS and the MB fluorescence signals were determined using TIRF microscopy (Nikon Eclipse Ti Inverted Microscope System).

### EV Membrane Protein Detection

EVs from cell lines or healthy donor and patient serum/plasma were diluted four times with PBS before use. 20 µL of EVs was added to each well on the biochip and incubated for 2 h at room temperature on a shaker. After washing with PBS, the biochip was blocked with 3% (w/v) BSA in PBS for 1 h at room temperature. Then, 20 µL of Alexa Fluor 647 labeled monoclonal antibody at a dilution of 1:500 in 1% (w/v) BSA in PBS was added to each well on the biochip and incubated for 1 h at room temperature. The biochip was washed with PBS and the fluorescence signals were determined using TIRF microscopy (Nikon Eclipse Ti Inverted Microscope System).

### TIRF Measurements and Image Analysis

TIRF microscopy (Nikon Eclipse Ti Inverted Microscope System) was used to record and analyze sample images. For GPC1 mRNA detection, 50 mW 561 nm lasers at 40% power were used to excite MBs labeled with Cy‐3. For GPC1 protein detection, 50 mW 640 nm lasers at 40% power were used to excite Alexa 647 labeled antibodies. Images were collected by an Andor iXon EMCCD camera with an Å≈100 lens and 200 ms exposure time. One hundred (10 × 10 array) images in each well were taken and MATLAB software (R2019B) was used to analyze the images. For image analysis (Figure S11, Supporting Information), all bright spots of the apparent signal in each TIRF image were identified. The background noise was eliminated by a Wavelet denoising method using MATLAB software to ensure that the area surrounding the spot was clear. The net fluorescence intensity of each spot was then calculated by subtracting the mean intensity of pixels in the spot from the mean intensity of pixels surrounding the spot. Proper cutoffs were employed which was based on the spot size. A histogram of net fluorescence intensities of all apparent spots was obtained. All collectives of spots falling within the threshold between 6 and 20 pixels in diameter, set through a user interphase, were identified as biomarker signals from EVs (all other signals were discarded as EV aggregates or noises). The average net total fluorescence intensity was defined as:

(2) $$TFI\;=\frac{\sum_{k=1}^{l}\sum_{j=1}^{m}\sum_{i=1}^{n}\left(FI_{in}^{i,j,k}-{\bar{FI}}_{lbg}^{j,k}\right)}{l}$$

where *n, m*, and *l* are the number of all the pixels in one bright spot, the number of the bright spots in one image and the number of all effective images, respectively. *FI_in_* is the pixel intensity inside each spot. ${\bar{FI}}_{lbg}$ is the mean value of the local smooth background that surrounds each bright spot. The image analysis procedure was executed automatically.

### Cryogenic Transmission Electron Microscopy

EVs from a PDAC patient serum were fractionated using a size exclusion qEV_70_ column. The qEV column was washed and equilibrated with PBS before use. Briefly, 0.5 mL serum was loaded onto the column and fractions were collected immediately using PBS as the elution buffer. A 0.5 mL of each fraction from 7 to 12 was collected. Then, fractions were concentrated to 0.1 mL using Amicon Ultra‐4 centrifugal filters with 10 kDa MWCO. Fractions were stored at −80 °C until use. For Cryo‐TEM analysis, 3 µL of EVs from Fraction 8 and Fraction 10 were applied to a specimen grid and immediately plunged into liquid ethane to rapidly form a thin layer of amorphous ice using a Thermo Scientific Vitrobot Mark IV system. The grid was transferred under liquid nitrogen to a Thermo Scientific Glacios Cryo‐TEM.

### Bead‐based Sorting of EV Subpopulations

The distinct EV subgroups from PANC‐1, MIA PaCa‐2, or HPDE6c7 cells were captured by a biotin‐conjugated antibody mixture, including i) anti‐ARF6/ANXA1 for MVs, ii) anti‐EGFR/EpCAM/GPC1 for tMVs, and iii) anti‐CD63/CD81/CD9 for Exos as previous shown. EVs sorted by biotin‐conjugated antibodies were further captured by Pierce Streptavidin Magnetic Beads (#88 816, ThermoFisher) at 4 °C overnight and then purified by magnetic stands. For Western blotting, the purified MV, tMV, and Exo subpopulations were lysed by NP‐40 lysis buffer (ThermoFisher) and 30 ng from the total protein was loaded. Blots were probed with the indicated primary antibodies followed by horseradish peroxidase‐conjugated secondary antibodies (Bio‐Rad). For RT‐qPCR analysis, the purified MV, tMV, and Exo subpopulations were individually lysed by the single‐cell lysis buffer (#4 458 235, ThermoFisher) and 30–50 pg RNAs were primed by either random hexamer (for total target mRNA, #SO142, ThermoFisher) or oligo dt (for poly(A)ed mRNA, #SO132, ThermoFisher) as suggested by the manufacturer. The Realtime PCR assay was performed with TaqMan Master Mixes by ViiA 7 Real‐Time PCR System (Thermo Fisher Scientific). The expression of target genes relative to the internal control gene was calculated by using the threshold cycle number (ΔΔCt).

### qRT‐PCR

The GPC1 mRNA levels in PANC‐1 EVs spiked healthy donor serum were quantified using qRT‐PCR. Total RNA from 150 µL of calibration samples made above was isolated using a plasma/serum RNA purification mini kit (#55000, Norgen Biotek Corp.) according to the manufacturer's instructions. cDNA was then synthesized from total RNA using a High‐Capacity cDNA reverse transcription kit (#4368814, Applied Biosystems). Subsequently, the target mRNA expression was quantified using a TaqMan Gene Expression assay (Hs00892476_m1, ThermoFisher Scientific) on a real‐time PCR instrument (Applied Biosystems).

### ELISA

The GPC1 protein level in PANC‐1 EVs spiked healthy donor serum was quantified using a human GPC1 ELISA kit (#ELH‐GPC1, RayBiotech). 100 µL of calibration samples were added to the ELISA plate and their target protein expression was detected according to the manufacturer's instructions.

### CA19‐9 Detection

The serum CA19‐9 protein level in clinical samples was measured using a ST AIA‐PACK CA19‐9 Assay (Cat# 0252761, Tosoh Bioscience, Inc.) in MSKCC. An enzyme immunoassay on the Cobas 410 instrument (Roche Diagnostic System, Basel, Switzerland) based on the monoclonal 1116‑NS‑19‑9 antibody (Fujirebio Diagnostics) was used to measure serum CA19‐9 protein levels in TVGH and CGMH‐Keelung. The threshold value of the assay was 37 U mL^−1^.

### Flow Cytometry

Flow cytometric experiments were performed by using Beckman Coulter Gallios flow cytometers (Brea, CA). PANC‐1, MIA PaCa‐2, and HPDE6c7 cells were stained using fluorescence‐labeled human GPC1 monoclonal antibodies and an isotype control. Absolute cell concentrations were obtained by quantitative flow cytometry using CountBright absolute counting beads (Invitrogen).

### Cell Viability Assay

PANC‐1, MIA PaCa‐2, and HPDE6c7 cells were plated at 5,000 per well in 96‐well plates and allowed to adhere overnight. The cells were then cultured in the absence or presence of different concentrations of Gemcitabine for 48 h. After treatment, the cell viability was measured by CellTiter96 MTS {3‐(4,5‐dimethylthiazol‐2‐yl)−5‐(3‐carboxymethoxyphenyl)−2‐(4‐sulfophenyl)−2H‐tetrazolium} proliferation assay (Promega, Madison, WI), according to manufacturer's instruction.

### Statistical Analysis

Data analysis of TIRF images from the ILN biochip assay was conducted using MATLAB R2019B. A total of one hundred (10 × 10 array) images were captured per well, and a customized MATLAB code was utilized to analyze these images (Figure S11, Supporting Information). All clinical samples were subjected to measurement in two wells on a biochip. Data were presented as mean ± SD. The statistical analysis was conducted using a two‐tailed Student's *t*‐test, unless specified otherwise. To assess the differential expression of GPC1 in PDAC patients, BPDs, and healthy donors, a Mann‐Whitney U test was applied. Diagnostic effectiveness was evaluated through receiver operating characteristic (ROC) curve analysis. Cutoff points were selected using Youden's index, which maximizes the sum of sensitivity and specificity. *p* values from distinct ROC curves were calculated using the MedCalc Statistical Software. For the prognosis study, the optimal cutoff value was determined based on the EV GPC1 expression and patient overall survival. Subsequently, patients were categorized into two groups according to the EV‐GPC1 expression cut‐off. Differences in overall survival between these two groups were assessed through Kaplan–Meier survival curves and the log‐rank test.

## Conflict of Interest

L.J.L. is a shareholder at Spot Biosystems Ltd. Other co‐authors declare no competing interests.

## Author Contributions

H.L. and C.‐L.C. contributed equally to this work. L.J.L., M.F., C.‐P.L., Y.‐S.S., C.‐H.W., and C.‐Y.F.H. designed the study and all experiments. L.J.L., E.R., H.L., and K.J.K. developed the technology. C.‐L.C. and H.L. performed cell culture, cell colocalization experiment, western blot, and qRT‐PCR. K.J.K., H.L., and L.T.H.N. optimized and fabricated ILN biochips and designed the M.B. X.W. developed the MATLAB code for image analysis. H.L., X.M., and W.C. performed statistical data analysis. H.L. and T.‐S.C. performed EV purification using TEI. L.T.H.N. performed cryo‐TEM analysis. X.Y.R. drew the schematic figures. M.F., C.‐P.L., C.‐H.W., S.D., T.L.R., T.‐S.C., G.R., M.S., R.T., Y.‐S.C. (CGMH), H.‐L.C., C.‐L.C., and H.‐L.C. provided patient samples. J.P., Y.M., W.‐N.T., and J.Z. assisted cell culture, NTA analysis, and EV purification using the qEV column. P.‐H.C., E.R., and Y.‐S.C. (NYCU) assisted with data collection. H.L. and L.J.L. wrote the paper with feedback from M.F., C.‐P.L., C.‐H.W., T.‐S.C., P.M.‐H.C., and C.‐Y.F.H.

## Supporting information

Supporting Information

## Data Availability

The data that support the findings of this study are available from the corresponding author upon reasonable request.
