# Supplementary material for: Extracellular Vesicular Analysis of Glypican 1 mRNA and Protein for Pancreatic Cancer Diagnosis and Prognosis
Source: Adv Sci (Weinh). 2024 Jan 10;11(11):2306373. doi: 10.1002/advs.202306373 (PMC10953589; doi:10.1002/advs.202306373)
Supplement: Supplementary file 1 — Supporting Information [file ADVS-11-2306373-s001.pdf]

ADVANCED SCIENCE
Open Access

Supporting Information

for *Adv. Sci.*, DOI 10.1002/advs.202306373

Extracellular Vesicular Analysis of Glypican 1 mRNA and Protein for Pancreatic Cancer Diagnosis and Prognosis

Hong Li, Chi-Ling Chiang, Kwang Joo Kwak, Xinyu Wang, Sital Doddi, Lakshmi V. Ramanathan, Sun M. Cho, Ya-Chin Hou, Tai-Shan Cheng, Xiaokui Mo, Yueh-Shih Chang, Hui-Lan Chang, Weiming Cheng, Wei-Ni Tsai, Luong T. H. Nguyen, Junjie Pan, Yifan Ma, Xilal Y. Rima, Jingjing Zhang, Eduardo Reategui, Yeh-Shiu Chu, Peter Mu-Hsin Chang, Pei-Hung Chang, Chi-Ying F. Huang, Cheng-Hsu Wang*, Yan-Shen Shan*, Chung-Pin Li*, Martin Fleisher* and L. James Lee**

Supplementary Materials for

Extracellular Vesicular Analysis of Glypican 1 mRNA and Protein for Pancreatic Cancer Diagnosis and Prognosis

This PDF file includes:

Supplementary Figure S1-11

Figure S1. ILN - TIRF setup.

Figure S2. ILN characterization

Figure S3. Effect of capture antibody on the ILN assay

Figure S4. Protein expression of GPC1, microvesicle markers, and exosome markers in MIA PaCa-2 and HPDE6c7 cells and cell derived EV subpopulations.

Figure S5. GPC1 protein co-localization with MV markers, CD40, Selectin and ARF6.

Figure S6. AUC/ROC curves for GPC1 Exo-mRNA and tMV-mProtein expression in Stage III/IV PDAC patients as single- or dual-biomarkers.

Figure S7. Dot charts, scatter plots, and ROC curves of GPC1 Exo-mRNA and tMV-mProtein expression as a dual biomarker for non-blinded validation PDAC patient samples from each hospital.

Figure S8. Dot charts, scatter plots, and ROC curves of GPC1 Exo-mRNA and tMV-mProtein expression as a dual biomarker for blinded validation samples from each hospital.

Figure S9. GPC1 Exo-mRNA and tMV-mProtein expression in MSKCC PDAC patients with low CA19-9 levels in blood.

Figure S10. CA19-9 levels in blood from late-stage pancreatic cancer patients undergoing chemotherapy.

Figure S11. ILN-TIRF image analysis approach.

Supplementary Table S1-S5

Table S1. Clinical characteristics of patients with BPD and IPMN for biomarker screening

Table S2. Clinical characteristics of CGMH PDAC patients undergoing chemotherapy

Table S3. Clinical characteristics of TVGH PDAC patients undergoing chemotherapy

Table S4. Clinical characteristics of NCKUH PDAC patients undergoing chemotherapy

Table S5. Summary of biomarkers reported in the literature for PDAC diagnosis and prognosis

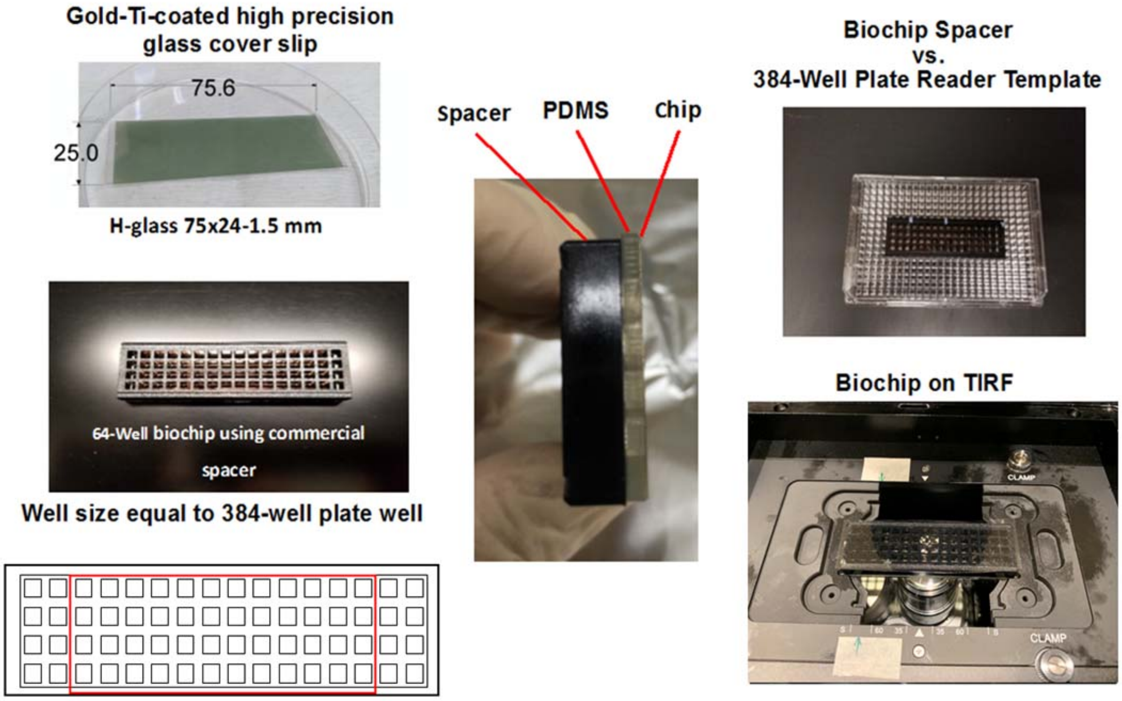

Figure S1. ILN-TIRF setup.

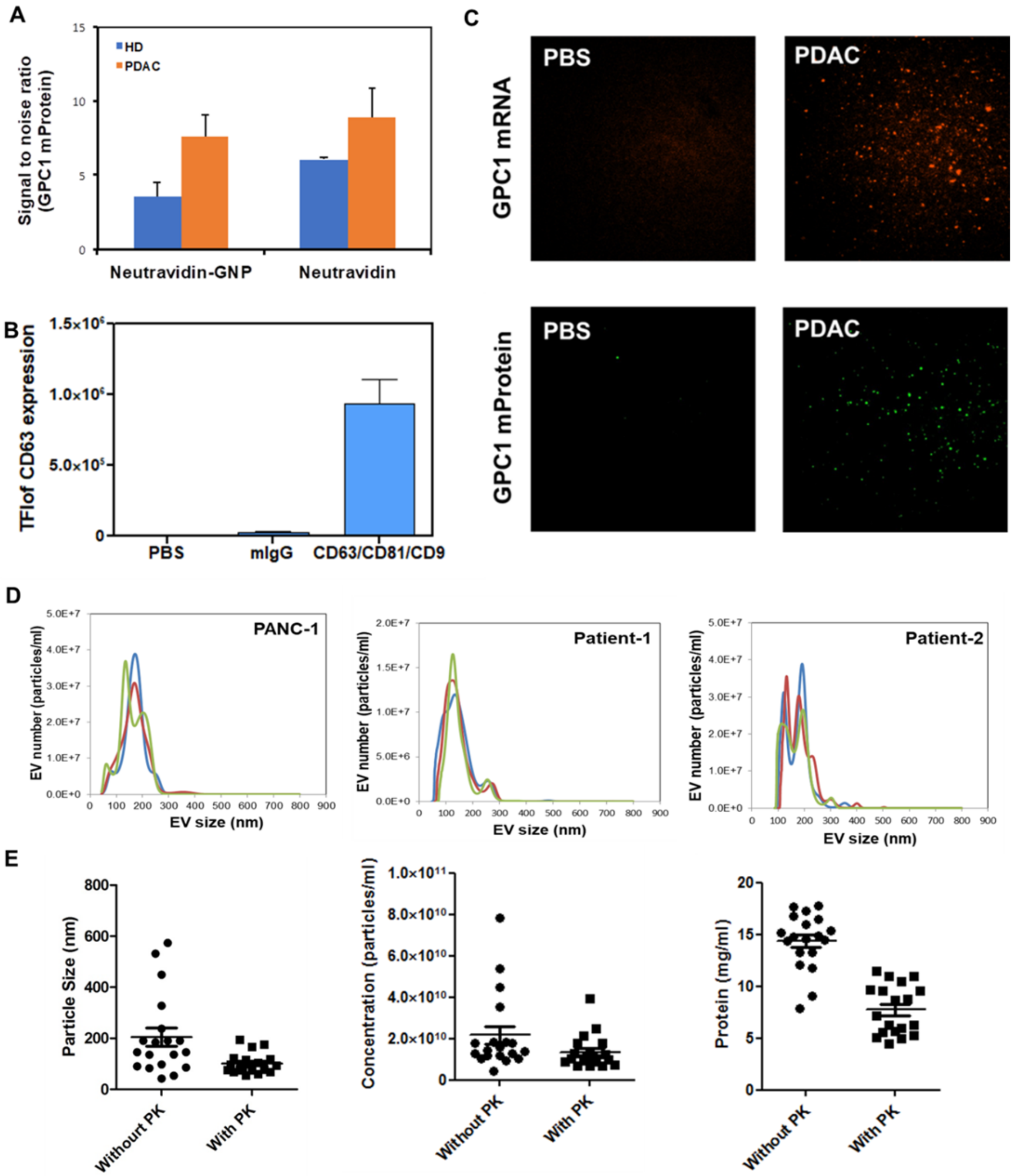

Figure S2. ILN biochip characterization. (A) Effect of neutravidin and neutravidin conjugated gold nanoparticle (Neutravidin-GNP) on TFI of GPC1 protein detection in HD and PDAC patients. **(B)** Non-specific binding of CD63 protein expression using the mIgG isotype as a control. Data were presented as means ± SD (*n* = 2 wells, each well with 100 images). **(C)** The TIRF images of minimal non-specific binding without (PBS) and with patient EVs on ILN biochips. **(D)** NanoSight nanoparticle tracking analysis of EVs from conditioned media of PANC-1 cells and different PDAC patients at low EV concentrations. **(E)** Effect of proteinase K (PK) on EV size, number, and protein contamination in PDAC patient serum (*n* = 20) from OSU. Data were presented as means (*n* = 3).

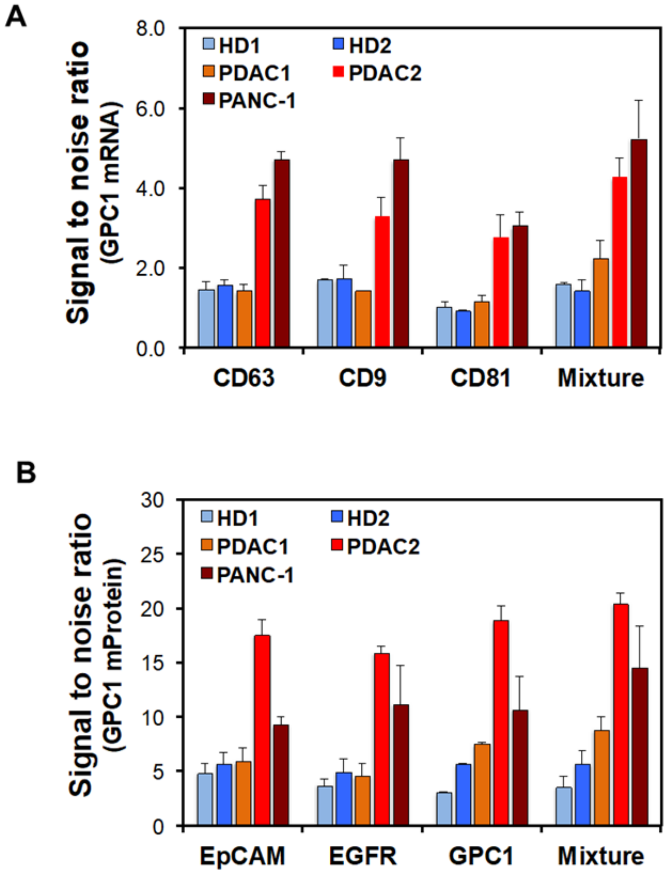

Figure S3. ILN characterization. **(A)** GPC1 mRNA and **(B)** GPC1 mProtein expression levels in EVs sorted by individual and mixed capture antibodies from PANC-1 cells, healthy donor (HD1, HD2) and PDAC patient (PDAC1, PDAC2) serum samples. All data were presented as means ± SD (n = 2 wells, each well with 100 images).

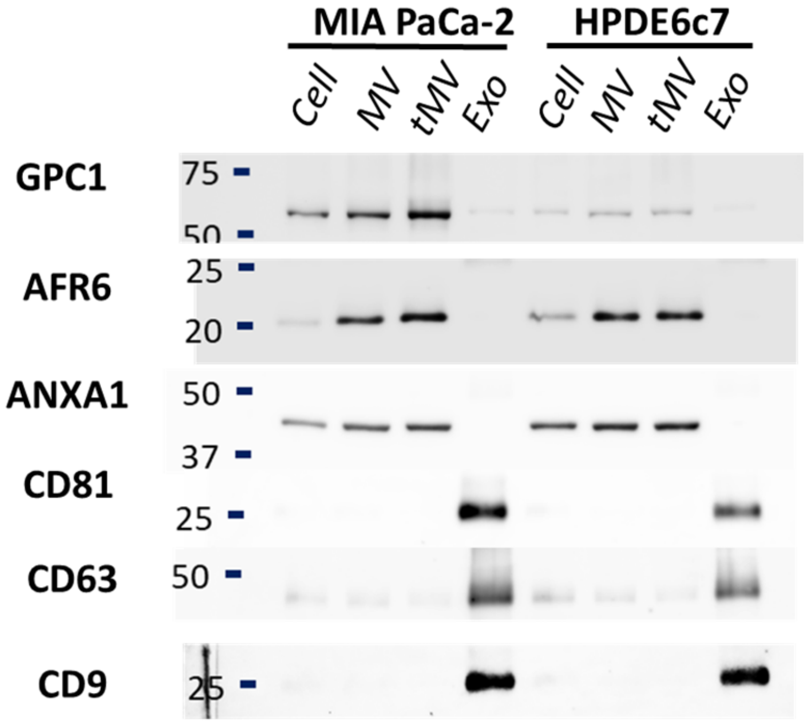

Figure S4. Protein expression of GPC1, microvesicle markers, and exosome markers in MIA PaCa-2 and HPDE6c7 cells and cell-derived EV subpopulations. The GPC-1 protein is highly expressed in MV and tMV fractions in EVs derived from PANC-1 and MIA PaCa-2 cell lines, but low in Exo fractions. Lower GPC-1 expression is found in non-cancerous cell line (HPDE6c7) and its EVs.

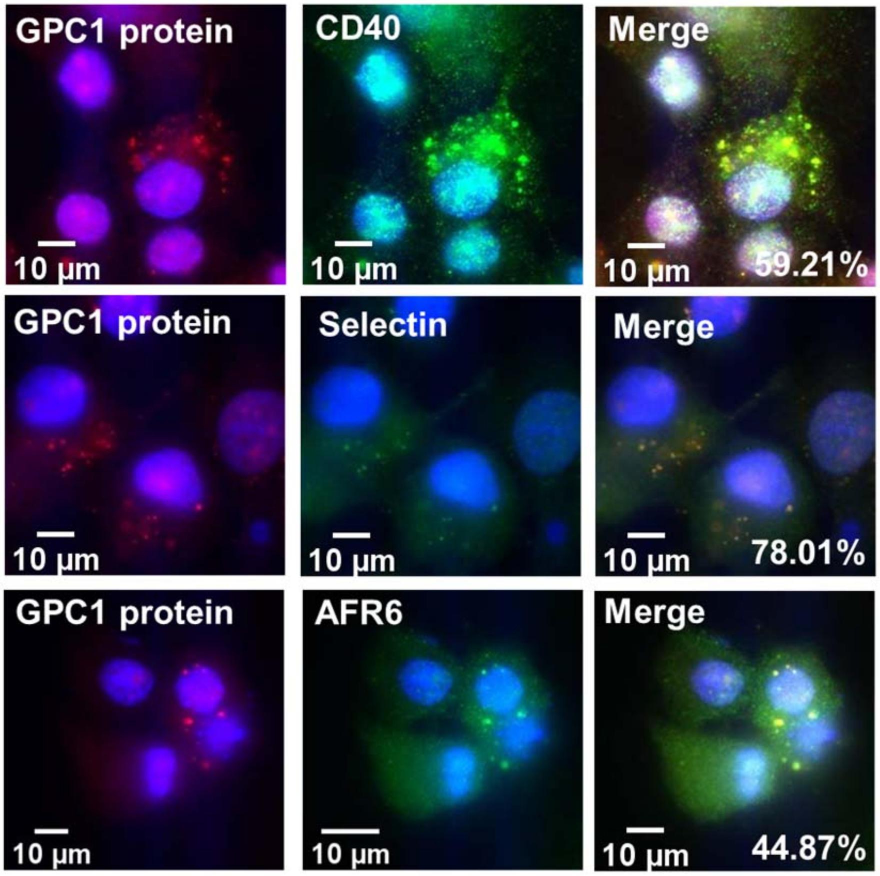

Figure S5. GPC1 protein co-localization with MV markers, CD40, Selectin, and ARF6.

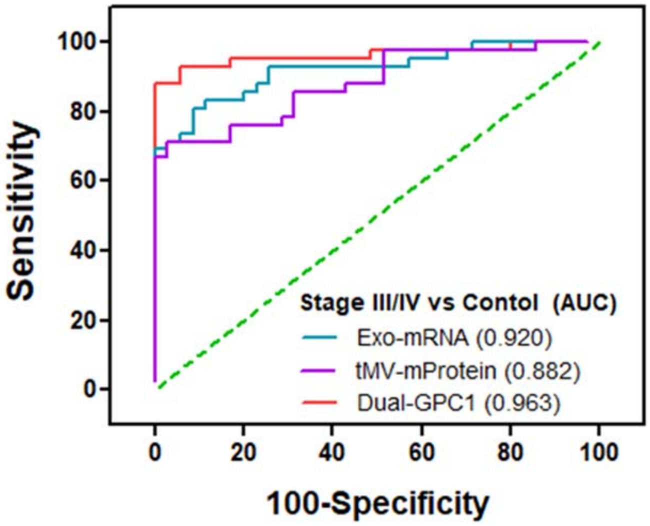

Figure S6. AUC/ROC curves for GPC1 Exo-mRNA and tMV-mProtein expression in Stage III/IV PDAC patients as single- or dual-biomarkers. PDAC patients from OSU compared to a control cohort consisted of HD and BPD samples.

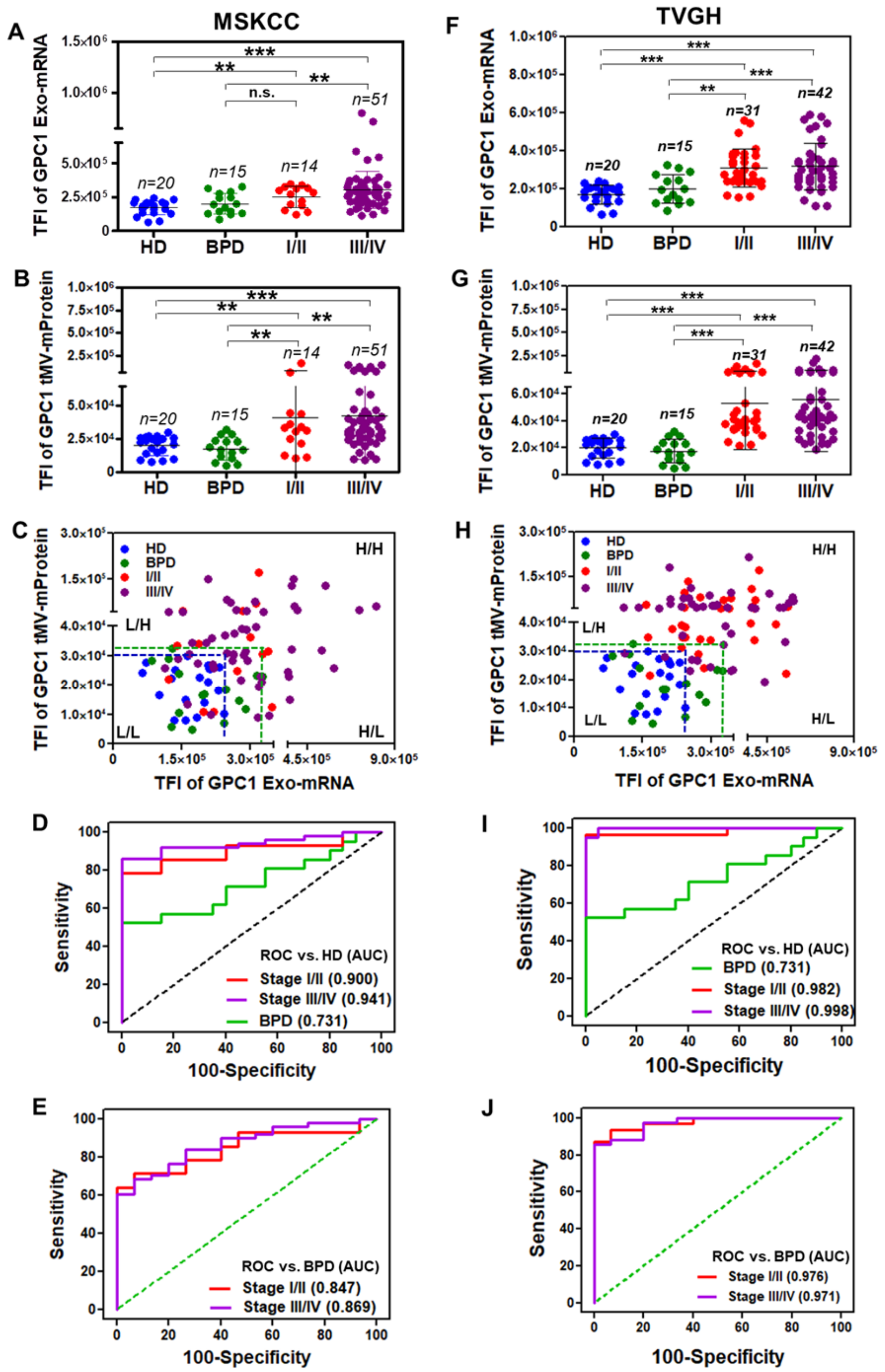

Figure S7. Dot charts, scatter plots, and ROC curves of GPC1 Exo-mRNA and tMV-mProtein expression as a dual biomarker for non-blinded validation PDAC patient samples from each hospital. (**A-E**) MSKCC in the US. (**F-J**) TVGH in Taiwan. All data were presented as means (n = 2 wells, each well with 100 images). Pairwise comparison *P* values were determined by the Mann-Whitney U test. $^{**}P < 0.01$, $^{***}P < 0.001$, n.s. not significant.

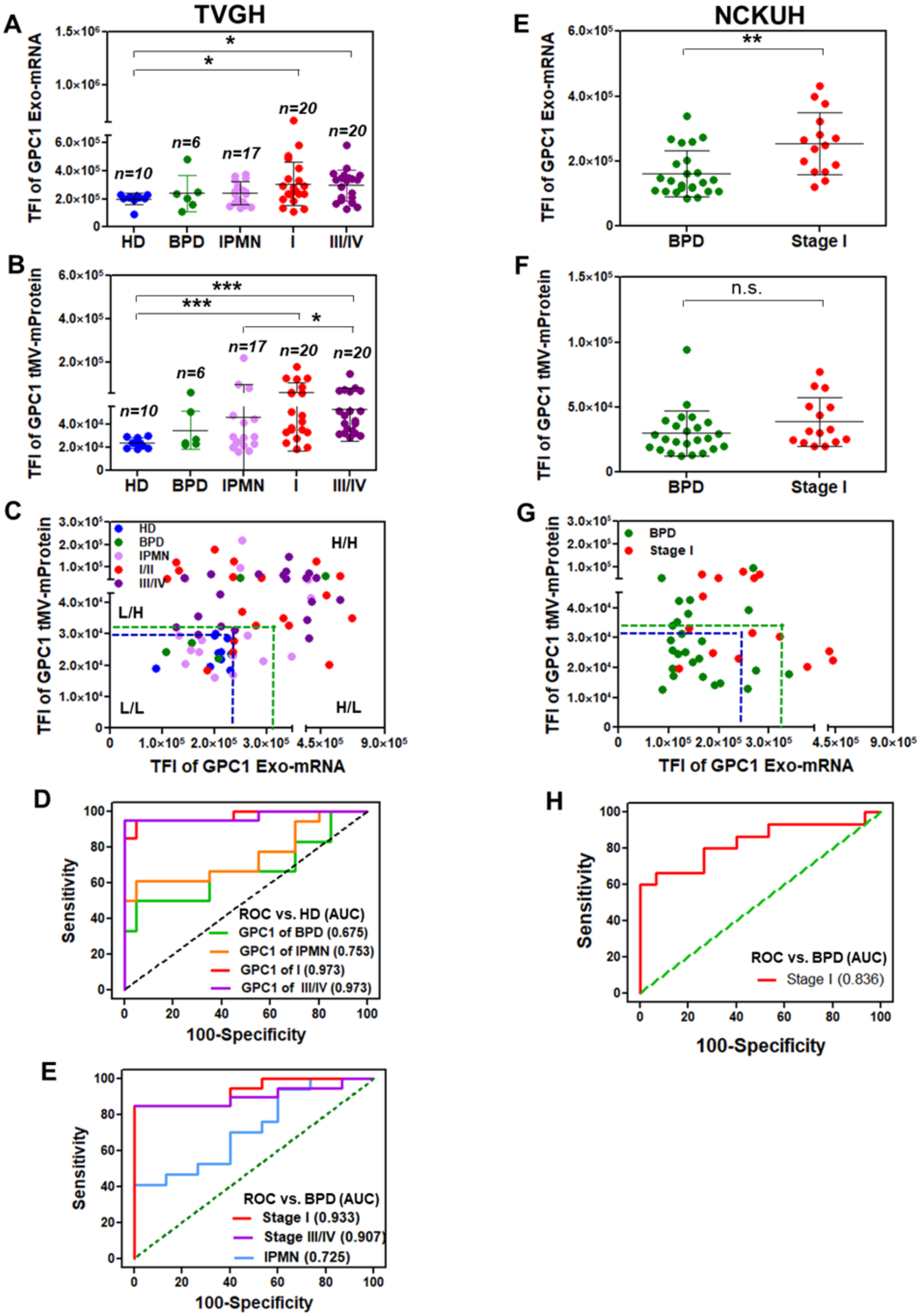

Figure S8. Dot charts, scatter plots, and ROC curves of GPC1 Exo-mRNA and tMV-mProtein expression as a dual biomarker for blinded validation samples from each hospital. (A-E) TVGH, **(F-J)** NCKUH. All data were presented as means (n = 2 wells, each well with 100 images). Pairwise comparison *P* values were determined by the Mann-Whitney U test. $*P < 0.05$, $**P < 0.01$, $***P < 0.001$, n.s. not significant.

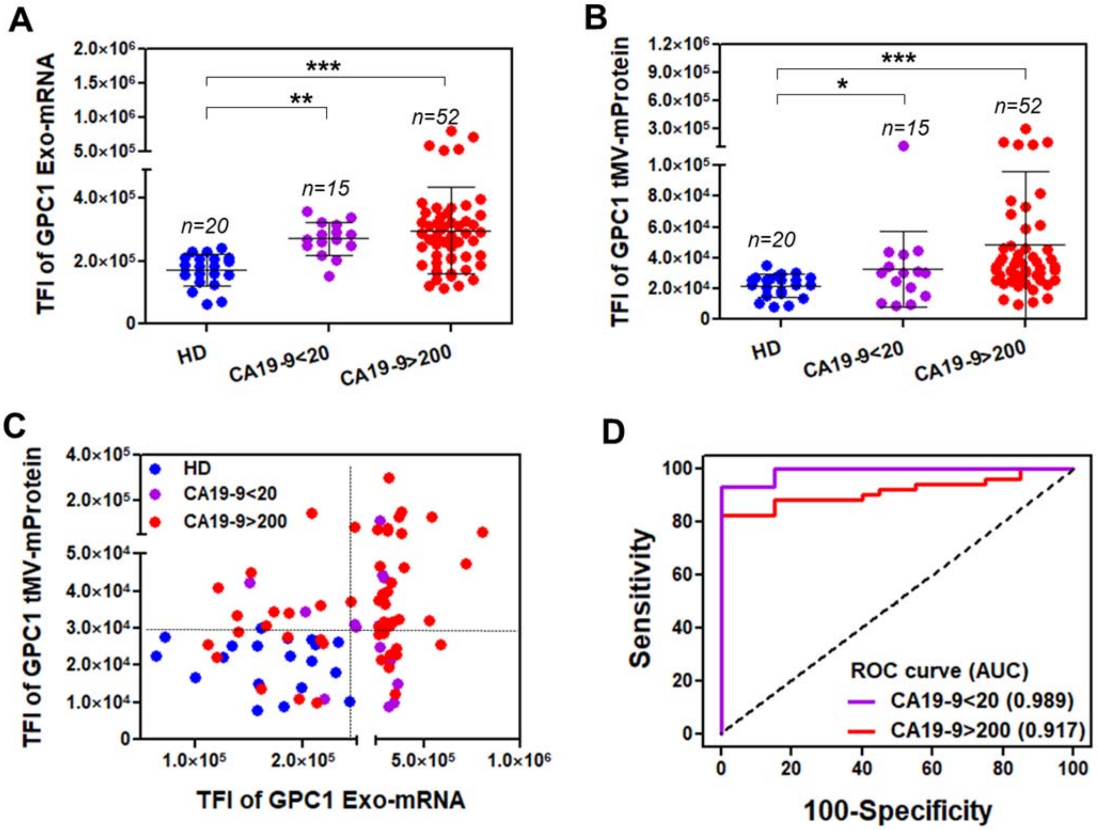

Figure S9. GPC1 Exo-mRNA and tMV-mProtein expression in MSKCC PDAC patients with low CA19-9 levels in blood. (**A** and **B**) GPC1 Exo-mRNA and tMV-mProtein expression for PDAC patients with low and high CA19-9. All data were presented as means (n = 2 wells, each well with 100 images). Pairwise comparison *P* values were determined by the Mann-Whitney U test. *$P < 0.05$, **$P < 0.01$, ***$P < 0.001$, n.s. not significant. (**C**) Scatter plot of GPC1 tMV-mProtein vs. GPC1 Exo-mRNA. (**D**) ROC curve for PDAC patients with high and low CA19-9 compared to healthy donors (HD).

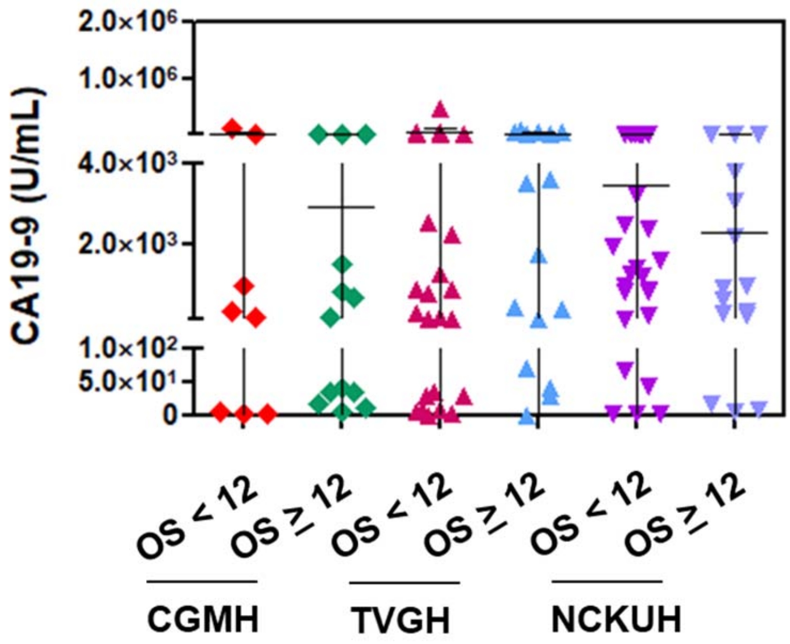

Figure S10. CA19-9 levels in blood from late-stage pancreatic cancer patients undergoing chemotherapy. CA19-9 levels of PDAC patients before chemotherapy (C0) with < 12 months) and ≥ 12 months overall survival (OS).

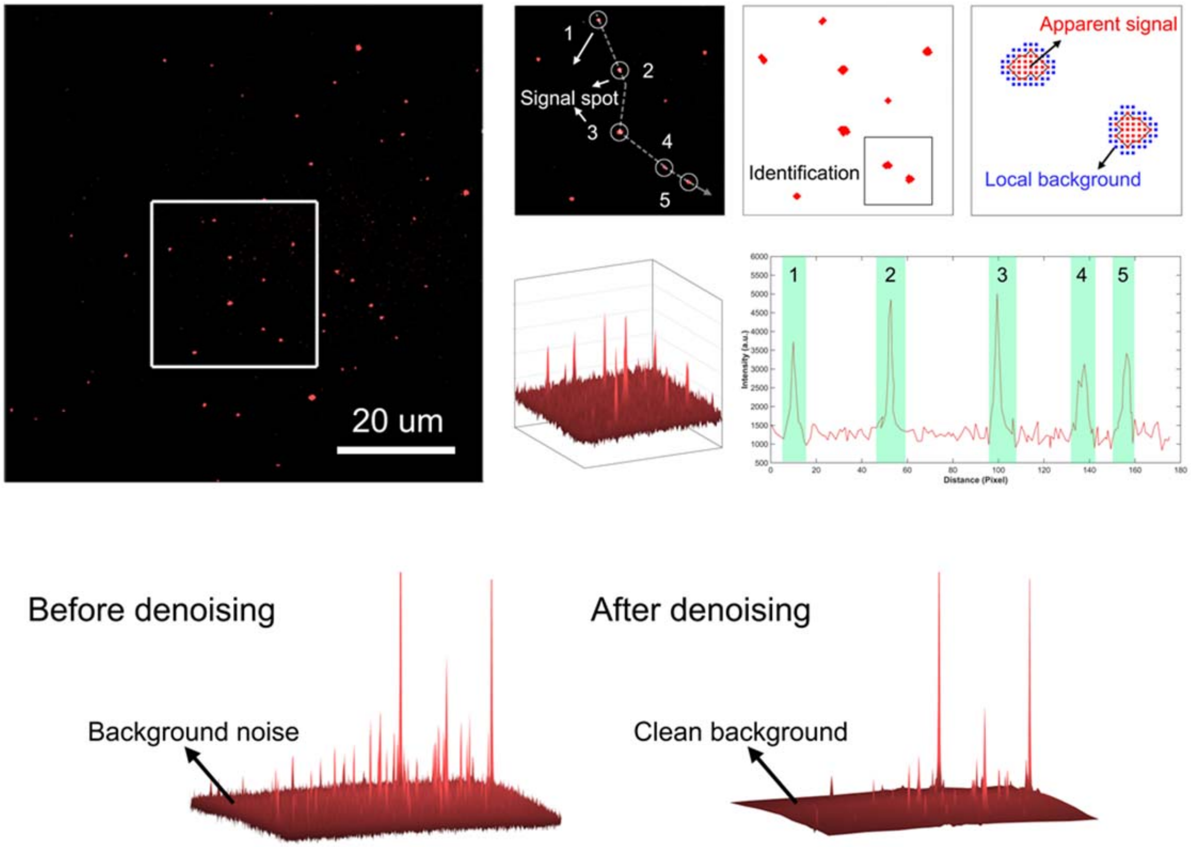

Figure S11. ILN-TIRF image analysis approach.

Table S1. Clinical characteristics of patients with BPD and IPMN for biomarker screening

Characteristics	BPD and IPMN samples (Biomarker screening)			
	Discovery	Blinded validation		
**Hospitals**	BPD (TVGH) (n=15)	BPD (TVGH) (n=6)	BPD (NCKUH) (n=24)	IPMN (n=17)
**Age (years)**				
Median (range)	54 (30-80)	67 (47-80)	59 (24-85)	66 (42-84)
**Gender, n (%)**				
Male	14 (93.3 %)	2 (33.3 %)	16 (66.7 %)	46 (53.3 %)
Female	1 (0.07 %)	4 (66.7 %)	8 (33.3 %)	27 (46.7 %)
**BPD and IPMN at diagnosis, n (%)**				
CP	12 (80.0 %)	2 (33.3 %)	11 (45.8 %)	-
SCA	3 (20.0 %)	4 (66.7 %)	6 (25.0 %)	-
AI	-	-	4 (16.7 %)	-
MPC	-	-	3 (12.5 %)	-
IPMN	-	-	-	17
**CA19-9 levels**				
Low (≤ 37)	13 (86.7 %)	5 (83.3 %)	9 (37.5 %)	14 (82.4 %)
High (>37)	2 (13.3 %)	1 (16.7 %)	5 (20.8 %)	3 (17.6 %)
ND	-		10 (41.6 %)	-

CP: Chronic pancreatitis, SCA: Serous cystadenoma, AIP: Autoimmune pancreatitis
MPC: Mucinous pancreatic cysts, ND: Not detected

Table S2. Clinical characteristics of CGMH PDAC patients undergoing chemotherapy

Lable	Age	Gender	PDAC stage	OS (Months)	Treatment regimens
P1	67	M	IV	8.0	Gemcitabine/cisplatin
P2	63	M	IV	5.33	Folfirinox
P3	53	F	IV	7.4	Gemcitabine/TS-1
P7	62	M	IV	6.6	Gemcitabine
P8	61	F	IV	7.9	Gemcitabine/cisplatin
P10	67	F	IV	7.6	Gemcitabine/cisplatin
P11	87	M	IV	2.17	Abraxan/Gemcitabine
P20	70	F	IV	4.5	Gemcitabine
P4	58	F	III	>32.3	Folfirinox
P5	58	F	III	28.7	Gemcitabine
P6	64	M	IV	> 28.3	Gemcitabine/cisplatin
P9	69	M	IV	13.1	Abraxan/Gemcitabine
P12	60	F	IV	> 15.6	Abraxan/Gemcitabine
P13	66	M	III	> 15.6	Abraxan/Gemcitabine
P14	41	M	III	> 14.7	Abraxan/Gemcitabine
P15	63	F	III	> 15	Abraxan/Gemcitabine
P17	64	F	IV	12	Abraxan/Gemcitabine
P18	58	M	IV	> 12.6	Abraxan/Gemcitabine
P21	71	M	IV	>12	Abraxan/Gemcitabine
P24	62	M	IV	> 17.7	Abraxan/Gemcitabine/Pembro
P25	67	F	III	> 24	Gemcitabine
P26	77	M	IV	>14.3	Abraxan/Gemcitabine

Table S3. Clinical characteristics of TVGH PDAC patients undergoing chemotherapy

Label	Age	Gender	PDAC Stage	OS (Months)	Treatment regimen	CA19-9
T1-0	69	M	IV	12.9	Gemcitabine/Tarceva/TS-1	70612
T2-0	69	M	IB	14.3	Gemcitabine/Tarceva	34080
T3-0	71	M	IV	15.8	Gemcitabine/Sutent	43000
T4-0	61	M	IV	12.8	Gemcitabine	6151
T5-0	71	M	IV	16.2	Gemcitabine/Tarceva/Xeloda x1	3600
T6-0	64	M	IV	18.2	Gemcitabine	5258
T7-0	71	M	IV	16.1	Gemcitabine+Tarceva	0.23
T8-0	46	M	IV	12.9	Gemcitabine/Oxaliplatin/Tarceva/Docetaxel/Cisplatin/Ufur	376
T9-0	53	M	IV	12.2	Gemcitabine/Tarceva	30.13
T10-0	69	F	IV	14.2	Gemcitabine/Tarceva	1730
T11-0	58	M	IV	15.1	Gemcitabine/MEK/TS-1/FOLFIRINOX	405
T12-0	66	M	III	19.8	Gemcitabine/Tarceva	3489
T13-0	72	F	III	15.9	Gemcitabine/Tarceva/FOLFIRINOX	42.57
T14-0	62	M	IV	32.1	Gemcitabine/GSK1120212/5-FU/ Leucovorin/Tarceva	113
T15-0	60	M	III	25.4	Gemcitabine/GSK1120212/Tarceva	71.23
T16-0	84	M	IV	19	Gemcitabine/Tarceva	45351
T17-0	50	F	IV	7.73	Gemcitabine/Tarceva	1.94
T18-0	78	F	IV	1.87	Gemcitabine/Tarceva	103
T19-0	51	M	IV	7.53	Gemcitabine/Tarceva	125
T20-0	80	M	IV	7.40	Gemcitabine/Tarceva	24820
T21-0	48	M	IV	6.23	Gemcitabine/Tarceva	25823
T22-0	66	M	IV	10.23	Gemcitabine/Tarceva	2204
T23-0	74	M	IV	3.5	Gemcitabine	136
T24-0	40	F	IV	6.7	Gemcitabine/Tarceva	4178
T25-0	79	F	IV	9.47	Gemcitabine/Tarceva	2534
T26-0	69	M	IV	7.4	Gemcitabine/Tarceva	35.4
T27-0	58	M	IV	6.13	Gemcitabine	30.78
T28-0	84	M	IV	8.73	Gemcitabine/Tarceva	1229
T29-0	59	F	IV	8.77	Gemcitabine/Tarceva	8.77
T30-0	66	M	IV	11.31	Gemcitabine	7097
T31-0	80	M	IV	3.23	Gemcitabine	10289
T32-0	74	M	IV	3.97	Gemcitabine/Tarceva	439380
T33-0	67	M	IV	9.2	Gemcitabine	9.28
T34-0	74	M	IV	4.43	Gemcitabine/Tarceva	742
T35-0	42	M	IV	4.03	Gemcitabine/Tarceva	4.03
T36-0	83	M	IV	3.47	Gemcitabine/Tarceva	30.8
T37-0	68	F	IV	10.2	Gemcitabine/Tarceva	868
T38-0	73	M	IV	3.4	Gemcitabine	269
T39-0	63	F	IV	8.633	Gemcitabine/Tarceva	6.91
T42-0	57	F	IV	8.93	Gemcitabine	852

Table S4. Clinical characteristics of NCKUH PDAC patients undergoing chemotherapy

Lable	Age	Gender	PDAC Stage	OS (Months)	Treatment regimens
U071	55	F	IV	13.1	Abraxane/Gemcitabine
U074	40	F	III	14.8	Abraxane/Gemcitabine
U078	66	F	IV	13.2	Abraxane/Gemcitabine
U080	62	F	IV	14.8	Abraxane/Gemcitabine
U081	59	M	IV	14.6	Abraxane/Gemcitabin
U082	80	F	I	> 16.7	Gemcitabine/Oxaliplatin/TS-1
U098	55	M	IV	12.2	Abraxane/Gemcitabine
U107	71	M	IV	> 16.1	Abraxane/Gemcitabine
U119	55	F	IV	> 15.2	Gemcitabine/Oxaliplatin/TS-1
U102	78	F	III	> 16.8	Gemcitabine/Oxaliplatin/TS-1
U130	38	M	IV	> 15.2	Abraxane/Gemcitabine
U132	55	M	IV	> 14.4	Gemcitabine
U135	67	F	IV	> 15.2	Abraxane/Gemcitabine +Add cisplantin
U158	65	F	III	> 14.1	Gemcitabine/Oxaliplatin/TS-1
U162	32	M	IV	> 12.6	Gemcitabine/Abraxane/TS-1
U192	64	F	IV	> 11.9	Abraxane/Gemcitabine
U106	60	F	IV	5.6	Abraxane/Gemcitabine
U086	62	M	IV	3.7	Abraxane/Gemcitabine
U089	51	M	IV	5.5	Abraxane/Gemcitabine
U090	64	F	III	9.5	Gemcitabine/Oxaliplatin/TS-1
U099	70	F	IV	5.5	Abraxane/Gemcitabine
U124	72	M	IV	8.0	Abraxane/Gemcitabine
U125	58	M	IV	10.8	Abraxane/Gemcitabine
U122	56	M	IV	7.3	Gemcitabine/Oxaliplatin/TS-1
U131	74	F	IV	6.5	Abraxane/Gemcitabin (3)+Gemcitabine
U136	67	F	IV	4.6	Abraxane/Gemcitabine
U141	85	M	I	10.4	Gemcitabine/Oxaliplatin/TS-1
U142	64	F	IV	5.3	Abraxane/Gemcitabine
U146	73	M	IV	11.2	Abraxane/Gemcitabine
U147	78	F	III	11.6	Gemcitabine/Oxaliplatin/TS-1
U150	56	M	IV	1.5	Abraxane/Gemcitabine
U153	87	F	IV	8.1	Gemcitabine + Gemcitabine/Oxaliplatin/TS-1
U154	84	F	III	9.2	Gemcitabine
U169	68	M	IV	8.1	Gemcitabine/Abraxane/Oxaliplatin
U168	63	M	III	7.9	Gemcitabine/Oxaliplatin/TS-1
U178	88	M	IV	3.7	Abraxane/Gemcitabine + Cisplantin
U182	69	M	IV	7.5	Gemcitabine/Abraxane/Oxaliplati
U184	63	M	IV	10.6	Abraxane/Gemcitabine
U189	67	M	IV	5.1	Gemcitabine/Abraxane/TS-1
U195	58	F	IV	11.4	Gemcitabine/Abraxane/TS-1
U201	57	F	II	11.4	Gemcitabine/Oxaliplatin/TS-1
U205	70	F	IV	11.4	Abraxane/Gemcitabine
U217	58	M	III	10.9	Gemcitabine/Oxaliplatin/TS-1
U222	82	M	IV	10.7	Abraxane/Gemcitabine

Table S5. Summary of biomarkers reported in the literature for PDAC diagnosis and prognosis

No.	EV purification	Method and EV biomarker	Outcomes	References
1	UC	GPC1 protein rich crExos by microbeads and flow cytometry	Levels of GPC1+ crExos were excellent for detecting PDAC, tumor burden and the survival of pre- and post-surgical patients, but results were not reproducible by other researchers	Melo et al., Nature, 2015
2	UC	GPC1 protein by LC/MS/MS and miRNA by RT-PCR	Exosomal GPC1 protein was not able to distinguish PDAC from non-patients well. Exosomal miRNA signature is superior to exosomal GPC1 or plasma CA 19-9 levels	Lai et al., Cancer Lett., 2017
3	UC	GPC1 protein in crExos by ELISA	GPC1 in crExos is good for distinguishing PDAC from healthy donors, but not from benign pancreatic diseases.	Frampton et al., Oncotarget, 2018
4	UC	EV protein signature by an advanced multiplexed plasmonic assay	GPC1 protein alone had a sensitivity of 82%, and a specificity of 52%, whereas a 5-protein $PDAC^{EV}$ signature including GPC1 provided a sensitivity of 86% and a specificity of 81%.	Yang et al., Sci. Transl. Med., 2017
5	TEI kit	Anti-CD63 captured GPC1+ exosomes by microbeads with flow cytometry	Diagnosis accuracy of GPC1+ exosomes was 63%. GPC1+ exosomes coupled with CA19-9 provideed 84% diagnostic accuracy for resectable PDAC.	Buscail et al., Transl. Oncol., 2019
6	UC	Exosomal GPC1 and CD82 by microbeads with flow cytometry	Exosomal GPC1 protein provided an AUC of 0.885 vs. healthy donors. Exosomal GPC1 and CD82 proteins combined with CA19-9 an AUC of 0.942 vs. healthy donors.	Xiao et al., Mol. Cancer Res., 2020
7	TEI-PK	GPC1 Exo-mRNA and tMV-mPrtoein by ILN assay	The AUC of Stage I/II PDAC was 0.960 for GPC1 Exo-mRNA and tMV-mProtein (dual-GPC1) expressions compared to benign pancreatic diseases.	Li et al., This Study
